# Study of red vine phenotypic plasticity across central-southern Italy sites: an integrated analysis of the transcriptome and weather indices through WGCNA

**DOI:** 10.3389/fpls.2024.1498649

**Published:** 2024-11-11

**Authors:** Angelo Sicilia, Clizia Villano, Riccardo Aversano, Ermanno Di Serio, Elisabetta Nicolosi, Filippo Ferlito, Angela Roberta Lo Piero

**Affiliations:** ^1^ Department of Agriculture, Food and Environment, University of Catania, Catania, Italy; ^2^ Department of Agricultural Sciences, University of Naples Federico II, Naples, Italy; ^3^ Council for Agricultural Research and Economics, Research Centre for Olive, Fruit and Citrus Crops, Acireale, CT, Italy

**Keywords:** *Vitis vinifera*, berry, transcriptomics, WGCNA, hub genes, GxE interaction

## Abstract

The grapevine (*Vitis* spp., family *Vitaceae*) is characterized by marked phenotypic plasticity. Its ability to withstand specific environmental conditions depends on the activation of highly coordinated responses resulting from interactions among genotypes (G) and environmental factors (E). In this study, the transcriptomes of commercially ripe berries of the Cabernet Sauvignon and Aglianico genotypes grown in open fields at three different sites in central-southern Italy (Campania, Molise and Sicily) were analyzed with RNA sequencing. These transcriptomic data were integrated with a comprehensive set of weather course indices through weighted gene co-expression network analysis (WGCNA). A total of 11,887 differentially expressed genes (DEGs) were retrieved, most of which were associated with the Aglianico genotype. The plants from the Sicilian site presented the greatest number of DEGs for both genotypes. Most of the weather course data (daily maximum air temperature, relative humidity, air pressure, dew point, and hours of sun radiation) were significantly correlated with the “lightcyan1” module, confirming WGCNA as a powerful method for identifying genes of high biological interest. Within this module, the gene encoding the ACA10 cation transporter was highly expressed in plants of both genotypes from Campania, where the lowest anthocyanin content was recorded. The transcriptome was also correlated with quality traits, such as total soluble solids and polyphenol content. This approach could lead to the identification of a transcriptomic profile that may specifically identify a genotype and its growing site and to the discovery of hub genes that might function as markers of wine quality.

## 1 Introduction

The phenotype of every organism is determined by the combination of its genotype (G), the environment (E) and the genotype-dependent response to different environments, or the genotype × environment (GxE) interaction (Grishkevich and Yanai, 2013; El-Soda et al., 2014). Phenotypic plasticity is the ability of organisms with the same basal genotype to express different phenotypes depending on the circumstances, and this ability has gained ample attention from researchers recently because of the challenges posed by climate change (Nicotra et al., 2010). The stability of crop growth and yields must be maintained over diverse and dynamic environments, and understanding how a genotype responds to and interacts with the environment is necessary to predict the effects of climate change on ecology and modern agriculture (Fournier-Level et al., 2011; Sasaki et al., 2015; Del Santo et al., 2018). The grapevine (*Vitis* spp., family *Vitaceae*) is a fruit crop used to produce food and beverages that is economically important worldwide. This crop is characterized by pronounced phenotypic plasticity, which often results in large variations in the metabolic composition of the berry (Gomez et al., 2024). Phenotypic plasticity is advantageous because it enables the production of a diverse range of wines from the same cultivar and facilitates the adaptation of existing cultivars to different growing regions (Keller, 2010; Dai et al., 2011). Hence, understanding the adaptability and phenotypic plasticity of vine cultivars is becoming fundamental to support the resilience of local viticultural systems and preserve the typical characteristics of their wines (Nicolosi et al., 2022).

In the Mediterranean region, a decrease in rainfall coupled with an increase in temperature are expected (IPCC, 2022), leading to both a shift in suitable areas to satisfy the specific thermal requirements of grapevine cultivars and a decline in soil water availability. Consequently, the combination of stressors and expected water scarcity will directly affect grape and wine quality (Duchêne, 2016; Bonfante et al., 2018). However, cultivated grapes possess a specific biological ability to adapt to changes in climate; this ability is attributed to the occurrence of a comprehensive reorganization of whole-genome expression, involving changes at the transcriptional, epigenetic and network levels. This adaptation might be best exemplified by the concept of *terroir*, which encompasses the combined influences of varietal attributes, climate, soil conditions, winemaking practices, and their multitude of interactions (Bonfante and Brillante, 2022; Iorizzo et al., 2023).

Previous investigations of grape GxE interactions revealed a high level of differentiation among vineyards according to their geographical origin (Anesi et al., 2015; Xie et al., 2017; Dal Santo et al., 2013, 2018). Dal Santo and coworkers investigated the phenotypic plasticity of grapevines by comparing the berry transcriptome of a single clone of the vegetatively propagated cultivar Corvina across eleven vineyards in three major wine production macro areas of the Verona region (Bardolino, Valpolicella, and Soave) (Dal Santo et al., 2013). Subsequently, Anesi et al. (2015) characterized the metabolome and transcriptome of berries of the Corvina variety cultivated in seven different vineyards in similar macrozones in the province of Verona (Garda Lake, Valpolicella and Soave). Both studies revealed a clear terroir-specific effect on the transcriptome and metabolome such that each vineyard could be distinguished by a unique profile of specific metabolites (Dal Santo et al., 2013; Anesi et al., 2015). More recently, the phenotypic plasticity and GxE interactions of two grapevine varieties were investigated by transcriptome analysis of berries from three central Italian locations (Bolgheri on the Tuscany coast, Montalcino in the Tuscany hills and Riccione on the Adriatic coast) (Dal Santo et al., 2018). The results confirmed the previous findings (Dal Santo et al., 2013), indicating that a) the transcriptomic plasticity of berries is underpinned by broad transcriptional reprogramming, b) within-cultivar diversity may modulate gene expression in response to environmental cues, and c) the location of the vineyard plays an important role in determining the performance of each genotype by enhancing qualitative traits related to wine aroma and color. A common characteristic of these studies is that the different environments were very close to each other in regions characterized by similar latitudes and altitudes.

Weighted gene co-expression network analysis (WGCNA) is a systems biology method that clusters genes with similar expression patterns into the same module according to the correlation between gene expression and the interrelatedness of life activities in plants. Therefore, WGCNA has been widely used to study the biological relationships between co-expression networks and phenotypic traits (Langfelder and Horvath, 2008). In recent years, WGCNA has been successfully used to identify hub genes and pathways involved in the response of several species, including poplar (Wang et al., 2023), melon (Shen et al., 2022), sugarcane (Tang et al., 2023), maize (Yu et al., 2023), lemon (Sicilia et al., 2024) and grape (Villano et al., 2023), to biotic and abiotic stress. In the grape study, the integration of metabolome and transcriptome data enabled the identification of 15 hub genes that are highly correlated with terpenoids, branched-chain amino acids and lipids, offering new insights into the regulation of aroma-related biosynthesis pathways in grape varieties used for high-quality wine production (Villano et al., 2023).

In this study, we explored the GxE interactions of two red grape varieties, the internationally renowned Cabernet Sauvignon and the indigenous Italian variety Aglianico. Aglianico is a traditional cultivar primarily grown in the central-southern Italian regions of Campania and Basilicata, whereas Cabernet Sauvignon, originally from France, is now grown extensively worldwide. The genomes of both varieties have been fully sequenced (Chin et al., 2016; Minio et al., 2017; Aversano et al., 2024) and served as important reference points for our investigation. The transcriptomes of fully ripe berries grown under open-field conditions at three different sites in central-southern Italy (Sicily, Molise and Campania) were analyzed using RNA sequencing (RNAseq). Furthermore, the relationship between the transcriptomic outcome and the growth site was revealed using WGCNA. The RNAseq results were correlated with several weather course parameters, which together constituted the specific climatic profile of the site, as well as with quality traits, including total soluble solid, anthocyanin and polyphenol contents. To our knowledge, this is the first time that transcriptomic data have been correlated with weather course parameters and quality traits in the study of grapes, leading to the discovery of hub genes that might function as markers of a particular growth site or quality trait.

## 2 Materials and methods

### 2.1 Site description, plant material and trial design

The research was conducted in three commercial vineyards located in three regions of central-southern Italy: Campania (CAM), Molise (MOL) and Sicily (SIC), each characterized by different altitudes and latitudes. The vineyard geo-references and elevations are detailed in Online Resource 1: **Supplementary Table S1**. For each region, two vineyards were chosen. Two black (*Vitis vinifera* L.) wine grape cultivars, Aglianico (A) and Cabernet Sauvignon (C), were grown at each vineyard. All the vines, which were grafted onto 140 Ruggieri rootstocks, were approximately fifteen years old and were planted between 2008 and 2010. In Campania and Molise, vines were planted in north−south rows, whereas in Sicily, they were planted on gentle slopes. In Molise, both cultivars were planted at a spacing of 1.20 m (within the row) × 2.90 m (between rows), and both were trained with the simple Guyot method, with a formation height of 60 cm. In Campania, Aglianico vines were spaced at 1.50 m (within the row) × 2.90 m (between rows), and Cabernet Sauvignon vines were spaced at 1.0 m (within the row) × 2.70 m (between rows); these vines were also trained using the simple Guyot method, with a formation height of 60 cm. In Sicily, Aglianico vines were spaced at 1.10 m (within the row) × 1.10 m (between rows), and Cabernet Sauvignon vines were spaced at 1.10 m (within the row) × 1.30 m (between rows). Both cultivars were bush trained at a height of 0.5 m, with two to six main branches, each of which was spur-pruned to one spur and two buds per spur. The experimental design was based on sampling three independent randomized plots of five rows, each containing 30 vines, to ensure representativeness. All the measurements were carried out on seven ‘index’ vines per block (Ferlito et al., 2023).

### 2.2 Sampling

Berries were collected at the commercial ripening stage during the 2021 growing season (8 September 2021 for the Campania site; 24 September 2021 for the Sicily site; and 20 October 2021 for the Molise site). At least ten berries were randomly selected from each index vine, avoiding those with visible damage and/or signs of pathogen infection, and pooled with berries from the other index plants from different blocks. For subsequent analyses, three independent pools (biological replicates) of 30 whole berries each were selected, immediately frozen in liquid nitrogen, and stored at -80°C. Each analysis included six experimental samples: Aglianico from Campania (A_CAM), Aglianico from Molise (A_MOL), Aglianico from Sicily (A_SIC), Cabernet Sauvignon from Campania (C_CAM), Cabernet Sauvignon from Molise (C_MOL) and Cabernet Sauvignon from Sicily (C_SIC).

### 2.3 Weather course measurements and indices

Weather course data were collected during the entire growing season by a recording climatic station located inside each vineyard. The daily maximum air temperature (DMAT), relative humidity (RH), air pressure (AP), dew point (DP), total precipitation (TP), evapotranspiration (ET) and hours of sun radiation (HSR) were measured. Weather course indices for WGCNA were calculated as follows: for DMAT, RH, AP and DP, the average values of the thirty days preceding the sampling date were used, whereas for TP, ET and HSR, the sum values of the thirty days preceding the sampling date were used.

### 2.4 Chemical analysis for quality traits

The total soluble solids (TSS), pH, titratable acidity (TA), polyphenol (PP) content, total anthocyanin (ANTH) content and maturity index (MI) were measured. Free-run juice was used to determine TSS measured by a digital refractometer with temperature correction (RX-5000 Atago Co., Ltd., Bellevue, WA, USA). The pH and titratable acidity were determined with an automatic titrator (Titrino model 798, Metrohm, Riverview, FL, USA) on 5.0 mL juice samples titrated against 0.1 M NaOH up to pH 8.2. TA was expressed as g/L of tartaric acid equivalents. The MI was estimated at harvest (soluble solids -°Brix and pH) using the following formula (Lana et al., 2021):


MI=√pH×°Brix

The PP content was determined using the Folin–Ciocalteu reagent assay (Singleton et al., 1999) and expressed as mg/kg of grapes. The total ANTH content was measured according to the methods of Lo Cicero et al. (2016) and expressed as mg/kg of fresh weight as reported by Ferlito et al. (2014), Ferlito et al. (2020). Principal component analysis (PCA) of these quality traits was carried out with the R function ‘*prcomp*’.

### 2.5 Statistical analysis

Analysis of variance (ANOVA) was performed with the R function ‘*aov*’ on the differences among quality traits for each genotype. A *post hoc* analysis based on the Tukey honestly significant differences (Tukey HSD) test was performed with the ‘*TukeyHSD*’ R function at significance levels (p values) of 0.05, 0.01 and 0.001.

### 2.6 RNA extraction

Ten berries from each biological replicate, which were kept frozen by the continuous addition of liquid nitrogen, were ground using a precooled mortar and pestle. Total RNA was isolated from ground whole berries, excluding seeds, using a slight modification of the method described by Japelaghi and coworkers (Japelaghi et al., 2011). Briefly, because grape berries are rich in water and secondary metabolites that might interfere with RNA yield and quality, a scale-up of the ground tissue was needed: in detail, 1 g of sample was added to 10 ml of extraction buffer. Next, a chloroform:isoamyl alcohol (24:1) extraction was performed three times. RNA degradation and DNA contamination were monitored by 1% agarose gel electrophoresis. The RNA purity and concentration were assayed using a NanoDrop spectrophotometer (Thermo Fisher Scientific, Waltham, MA, USA) (Russo et al., 2021; Strano et al., 2014). Before sequencing, RNA integrity was assessed using the Agilent Bioanalyzer 2100 system (Agilent Technologies, Santa Clara, CA, USA) (Santoro et al., 2022).

### 2.7 Library preparation, clustering and sequencing

Library preparation, clustering and sequencing were performed by Novogene Co., Ltd., UK (25 Cambridge Park, Milton Road, Cambridge, CB4 OFW, United Kingdom). One µg of RNA was used as input material for library preparation (eighteen libraries: three biological replicates × two varieties × three sites). The sequencing libraries were generated using the NEBNext^®^ Ultra™ RNA Library Prep Kit for Illumina^®^ (New England Biolabs, Ipswich, MA, USA), as reported in Sicilia et al. (2019). After cluster generation, the library preparations were sequenced on the Illumina HiSeq2000 platform to generate paired-end reads with a size of 2×150 bp. Raw reads in fastq format were first processed using in-house Perl scripts (Novogene Co., Ltd., UK). Clean data were obtained by removing reads containing adapters, reads containing poly-N and low-quality reads (Q score below 5 for more than 50% of the bases) (Online Resource 2: **Supplementary Table S2**). At the same time, the Q20, Q30, GC content and sequence duplication level of the clean data were calculated. All the downstream analyses were based on high-quality clean data.

### 2.8 *De novo* assembly and gene functional annotation

*De novo* transcriptome assembly was performed with Trinity software (version 2.6.6) with the parameters min_Kmer_Cov=3 and min_glue=4 (Grabherr et al., 2011). Hierarchical clustering was carried out with Corset (version 4.6) to remove redundancy (parameter -m 10) so that the longest transcript of each cluster was selected as the Unigene (Davidson and Oshlack, 2014). The assembly assessment and gene prediction were evaluated by comparing the Unigenes to the set of *Embryophyta* genes using the BUSCO quality assessment tool coupled with the OrthoDB (version 9.0) database of orthologues (Simão et al., 2015). The gene functional annotation was obtained using seven different databases: National Centre for Biotechnology Information (NCBI) non-redundant protein sequences (Nr, Diamond software, version 0.8.22, e-value threshold 1e-5) (Buchfnk et al., 2015), NCBI non-redundant nucleotide sequences (Nt, NCBI blast software, version 2.9.0, e-value threshold 1e-5), Protein Family (Pfam, hmmscan software, HMMER version 3.1, e-value threshold 0.01) (Finn et al., 2011), Cluster of Orthologous Groups of Proteins (KOG/COG, Diamond software, version 0.8.22, e-value threshold 1e-5) (Buchfnk et al., 2015), SWISS-PROT (Diamond software, version 0.8.22, e-value threshold 1e-5) (Buchfnk et al., 2015), Kyoto Encyclopedia of Genes and Genome (KEGG, Diamond and KAAS software, version 0.8.22, e-value threshold 1e-5) (Buchfnk et al., 2015; Moriya et al., 2007) and Gene Ontology (GO, blast2GO software, version b2g4pipe_v2.5, e-value threshold 1e-6) (Götz et al., 2008). The Unigene coding sequences were aligned to the *Vitis vinifera* genome PN40024 v4 using the Grapedia BLAST tool (https://grapedia.org) PN40024.v4.1_REF_prot database with an e-value threshold of 1e-5, followed by annotation as described by Grimplet et al. (2012). The annotated sequences not corresponding to the plant species were filtered out.

### 2.9 Quantification of gene expression and differential expression analysis

The gene expression level was estimated with RSEM software (version 1.2.28) by mapping each clean read back onto the assembled transcriptome, and the read counts for each gene were obtained from the mapping results (Li and Dewey, 2011). Furthermore, the read counts of each gene were used as input data for DESeq2 (version 1.26, padj ≤ 0.05) to obtain differentially expressed genes (DEGs) (Santoro et al., 2022). Six comparisons were made to identify the set of DEGs of each variety at the three sites (A_SIC vs. A_CAM; A_MOL vs. A_CAM; A_SIC vs. A_MOL; C_SIC vs. C_CAM; C_MOL vs. C_CAM; and C_SIC vs. C_MOL). An adjusted p value cut-off of 0.05 and a |log_2_fold change| (log_2_FC) threshold of 1 were the criteria used to filter significantly up- and downregulated genes. A correlation analysis was performed to demonstrate experimental repeatability and to reveal differences in gene expression among samples. PCA was performed using R language, considering the read counts of each sample as input data, including the biological replicates.

### 2.10 qRT−PCR validation

Total RNA (2.5 µg) was reverse transcribed using a SuperScript™ Vilo™ cDNA synthesis kit from Thermo Fisher Scientific according to the manufacturer’s instructions. To validate the reliability of RNAseq in determining gene expression levels, real-time qRT−PCR was performed for 10 random DEGs using PowerUp SYBR Green Master mix from Thermo Fisher Scientific and the Rotor-Gene Q 2plex detection system (Qiagen, Venlo, Netherlands) (Online Resource 3: **Supplementary Table S3**). All the genes were normalized to *Vitis vinifera* ubiquitin-60S ribosomal protein L40-2 (LOC100253716), which is a suitable housekeeping gene (Cheng et al., 2021b). All reactions were performed in duplicate, and the fold change values were calculated using the 2^−ΔΔCT^ method.

### 2.11 GO and KEGG enrichment analyses

Both GO and KEGG enrichment analysis of DEGs separated for each variety were performed using the ShinyGO V0.80 online tool (Ge et al., 2020). To define the genotype-specific transcriptomic response at each site, fragments per kilobase million (FPKM) values were used to identify genes specifically expressed at each site by each genotype. Six different gene lists were constructed (A_CAM, A_MOL, A_SIC, C_CAM, C_MOL, and C_SIC) by filtering genes that were expressed by a specific genotype at the site (FPKM ≠ 0) and had FPKM values at the other sites equal to zero. Genes of interest (expressed in only one site/genotype) were selected from the ten highest FPKM values in each cluster list.

### 2.12 WGCNA

A co-expression analysis of the gene expression data, weather course indices and quality traits was conducted. The co-expression analysis was performed using the Weighted Gene Co-expression Network Analysis (WGCNA) package in R (Langfelder and Horvath, 2008) to obtain hierarchical clustering and identify co-expressed genes (hub genes). Specifically, an adjacency matrix was created using the FPKM values of all the DEGs. The pickSoftThreshold() function was used to determine the optimal soft-thresholding power (Langfelder and Horvath, 2008). For each analysis, the lowest power for which the scale-free topology fit index was 0.90 was used (Online Resource 4: **Supplementary Figure S1**). The specific WGCNA parameters were set as follows: Soft powers β = 20 were selected using the function pickSoft Threshold; WGCNA ‘mergeCutHeight’ was set at 0.25. The adjacency matrix was transformed into a topological overlap matrix (TOM) and the corresponding dissimilarity matrix (1-TOM). Afterwards, a hierarchical clustering dendrogram of the 1-TOM matrix was constructed to classify genes with similar expression levels into different gene co-expression modules. Modules were merged by using a criterion of MEDissThres = 0.25. Finally, the relationships between each module and either the weather course indices or quality traits that were significantly different among sites for each genotype were estimated by Pearson’s correlation using the module eigengene values. Modules with high correlation coefficients and a correlation padj ≤ 0.05 were selected for subsequent analysis.

## 3 Results

### 3.1 Weather course data

The weather course indices obtained as described in the Materials and Methods section are reported in **Table 1**. The highest DMAT, AP, DP, ET and HSR values were recorded in Campania, as well as the lowest values of RH and TP. As expected, the highest TP was recorded in Sicily because of the seasonal rainfall pattern that is typical of the eastern slopes of Mt. Etna, where the vineyards were located. In addition, the lowest ET value was also recorded in Sicily. Finally, the highest RH and lowest DMAT, AP, DP and HSR values were recorded at the Molise site, thus indicating opposite weather conditions compared with those at the Campania site.

**Table 1:** Climatic indices measured at the three sites.

Experimental site	Parameter						
	DMAT (°C)	RH (%)	AP (Bar)	DP (°C)	TP (mm)	ET (mm)	HSR (hours)
CAM	31.5	64.9	996.4	16.7	23.9	122.6	308
SIC	29.1	66.2	968.9	15.4	167.5	23.4	252
MOL	19.5	81.1	949.9	10.2	80.3	42.6	186

DMAT, daily maximum air temperature; RH, relative humidity; AP, air pressure; DP, dew point; TP, total precipitation; ET, evapotranspiration; HSR, hours of sun radiation. Measurements from the thirty days preceding the sampling date were used; the DMAT, RH, AP and DP values were the average values over thirty days, and the TP, ET and HSR values were the sum values over thirty days.

### 3.2 Quality traits

**Figure 1:**
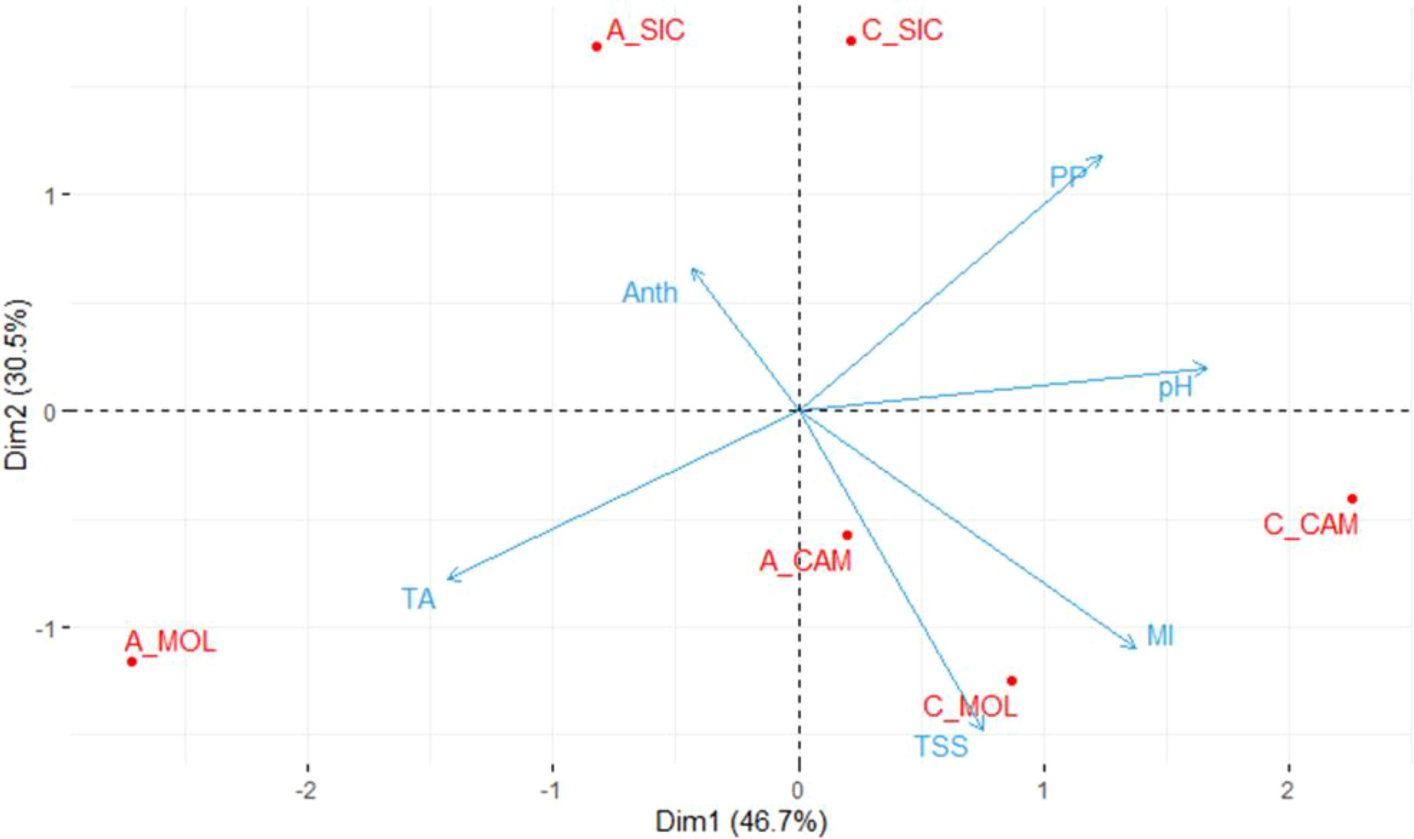
Principal component analysis (PCA) of the samples considering the quality traits. The average values of three biological replicates per sample were used. TSS, Total soluble solids; pH; TA, titratable acidity; PP, polyphenols; ANTH, anthocyanins; MI, maturity index; A, Aglianico; C, Cabernet Sauvignon; CAM, Campania; MOL, Molise; SIC, Sicily.

**Table 2** shows the mean values of the quality parameters of grape berries at the commercial ripening stage at the three sites. On average, Cabernet Sauvignon had a higher PP content than that of Aglianico at all three sites and the highest ANTH content, except at the CAM site, where the lowest value was recorded. Moreover, both Cabernet Sauvignon and Aglianico presented higher contents of TSS at the CAM and MOL sites than at the SIC site. Aglianico presented a significantly greater TA at the MOL site (**Table 2**). Interestingly, at all three sites, the Cabernet Sauvignon genotype exhibited significantly higher MI values than those of the Aglianico genotype (**Table 2**). **Figure 1** shows a PCA in which all the measured qualitative traits (PP, pH, TSS, TA and ANTH) were used as the variables. A total of 77.2% of the variability was explained by the first two components (dim1 and dim2), and the three variables that best differentiated the samples were TA, pH and PP. Interestingly, both the genotypes cultivated in Sicily clustered separately from the same genotypes cultivated at the other two sites. PP, TSS and MI were the traits that best separated the Sicilian site from the other sites. In CAM and MOL, the two genotypes were similar in terms of quality traits (TSS and MI), except for A_MOL, which had higher TA values than those of A_CAM, C_MOL and C_CAM.

**Table 2:** Quality traits measured at the three sites for both genotypes.

Genotype	Site	Parameter					
		TSS (°Brix)	pH	TA (g*L^-1^)	PP (mg/kg)	ANTH (mg*L^-1^)	MI
AGL	SIC	20.04 ± 0.73a	3.71 ± 0.13a	7.5 ± 0.01a	46.75 ± 7.16a	117.73 ± 20.27a	38.62 ± 1.60a
	CAM	23.22 ± 0.38b	3.63 ± 0.05a	7.9 ± 0.04a	45.59 ± 1.24a	109.14 ± 5.25a	44.26 ± 0.57c
	MOL	22.32 ± 0.88b	3.34 ± 0.08a	10.8 ± 0.41b	36.92 ± 7.03a	119.45 ± 36.63a	40.83 ± 2.08b
CAB	SIC	21.66 ± 0.98a	3.74 ± 0.05a	7.5 ± 0.00a	55.01 ± 12.14a	151.49 ± 34.37b	41.92 ± 1.78a
	CAM	22.99 ± 0.89ab	4.22 ± 0.37a	7.5 ± 0.01a	51.67 ± 5.67a	92.85 ± 15.49a	47.29 ± 3.72a
	MOL	24.45 ± 0.10b	3.79 ± 0.20a	7.6 ± 0.02a	43.49 ± 3.45a	133.94 ± 21.63ab	47.65 ± 1.44a

The average values of three replicates and the relative standard deviations are reported. Different letters indicate significant differences in each parameter among sites for each genotype. For the letters, ANOVA was performed. Tukey’s HSD test was performed at significance levels (p values) of 0.05, 0.01 and 0.001. TSS, total soluble solids; pH, pH; TA, titratable acidity; PP, polyphenols; ANTH, anthocyanins; MI, maturity index.

### 3.3 Transcript assembly and functional annotation

**Figure 2:**
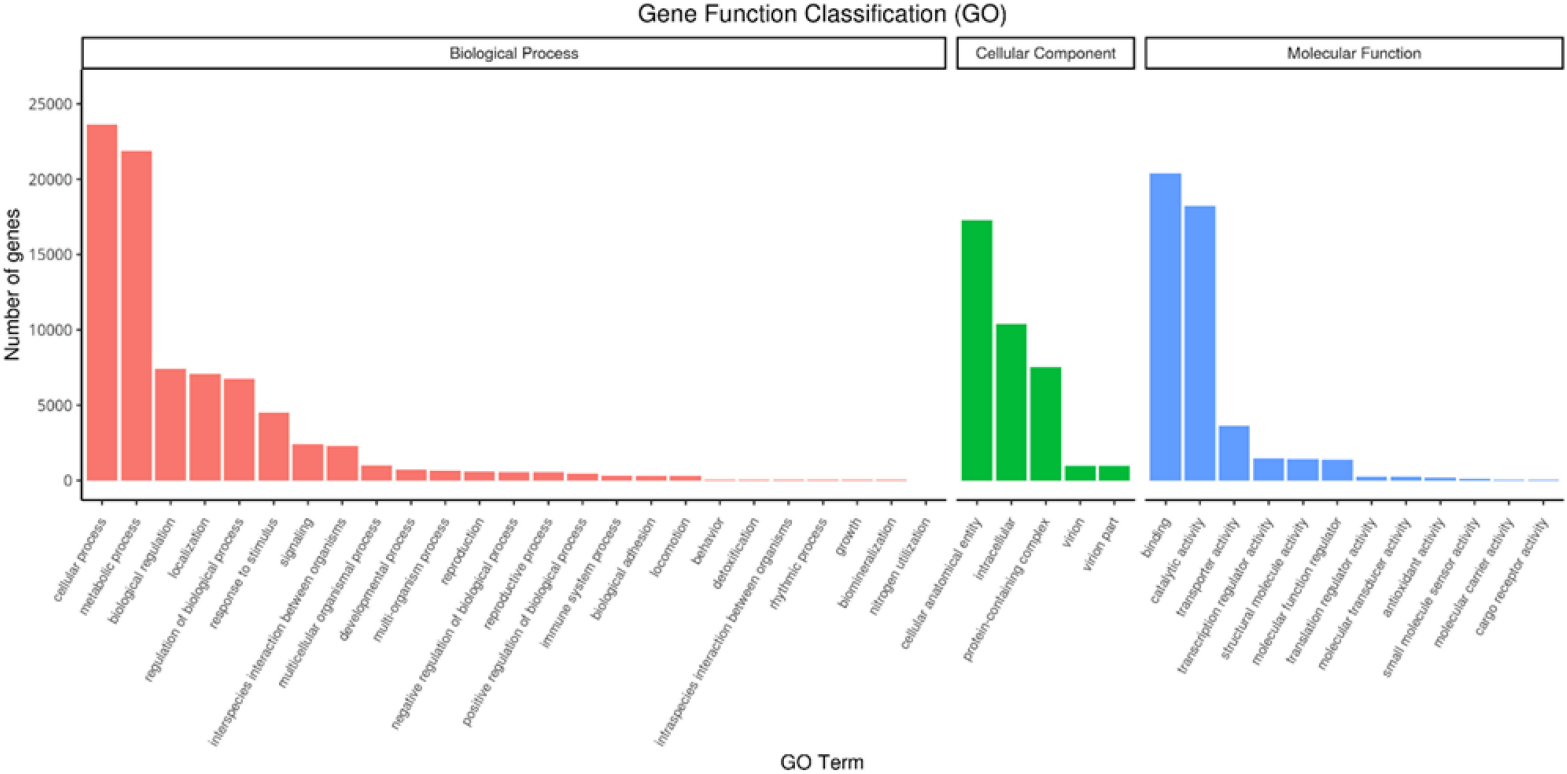
Unigene Gene Ontology (GO) functional annotation. The GO terms are grouped into three main categories: biological process, cellular component and molecular function.

**Figure 3:**
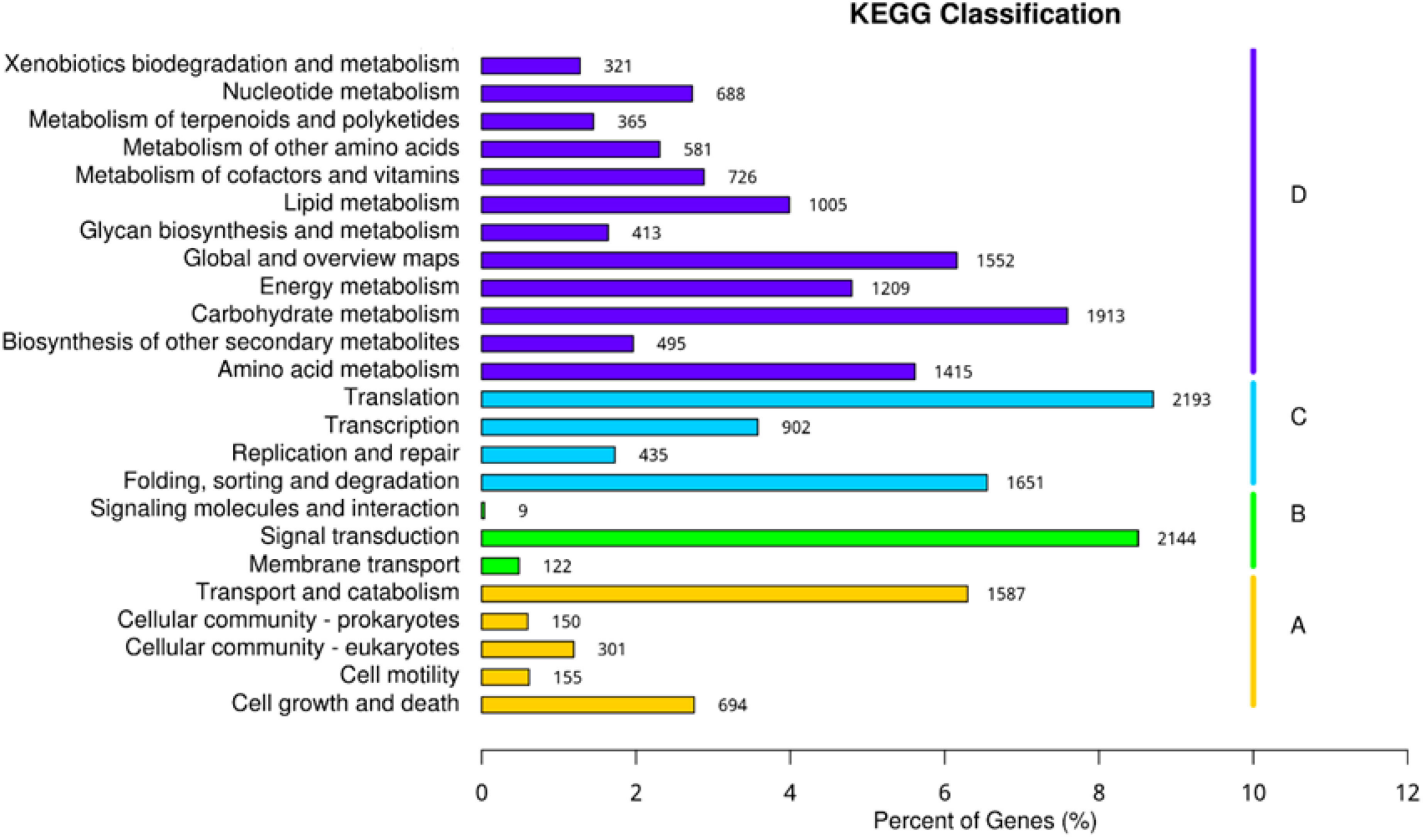
Unigene KEGG functional classification. **(A)** Cellular processes; **(B)** environmental information processing; **(C)** genetic information processing; **(D)** metabolism.

RNAseq was used to comprehensively identify the transcriptional profiles of Aglianico and Cabernet Sauvignon berries at the three sites in central-southern Italy. After library sequencing, the raw reads were filtered to remove adapter-based or poor-quality reads. A total of 598 million clean reads, representing 98.3% of the total reads, were obtained, with a Q30 value and a GC content of 91.54% and 46.62%, respectively (**Table 3**). The clean read *de novo* assembly yielded 226,498 transcripts and 97,442 Unigenes with N50 lengths of 2753 bp and 1881 bp, respectively (**Table 3**), which is consistent with previously reported N50 values (Santoro et al., 2022, 2023; Sicilia et al., 2022) and indicates good contiguity of the transcriptome. To assess assembly consistency, filtered unique reads were mapped back to the reconstructed transcriptome, and the average read mapping rate determined using Bowtie2 alignment software was 77.3% (mean value between 75.25 and 78.43) (**Table 3**). Among the 1440 groups searched with BUSCO, 63.1% (909 groups) were complete single-copy orthologues (**Table 3**). Functional annotation of the Unigenes was conducted by performing BLAST searches against public databases, such as the NCBI, Pfam, KOG/COG, SWISS-PROT, KO, and GO databases (**Table 4**). A total of 94,058 Unigenes were annotated in at least one database, corresponding to 96.52% of the total Unigenes. Among them, 64,056 (65.73%) and 87,354 (89.64%) Unigenes showed identity with the sequences in the Nr and Nt databases, respectively. The distributions of Unigenes homologous to the sequences in the KO, SWISS-PROT, Pfam, GO, and KOG databases were 21.61%, 45.03%, 41.87%, 41.87% and 18.50%, respectively (**Table 4**). With respect to the GO annotation, most of the Unigenes were annotated as “cellular process” (GO:0009987), “metabolic process” (GO:0008152), “biological regulation” (GO:0065007), “localization” or “regulation of biological process” (GO:0050789) within the Biological Process category. “Cellular anatomical entity” (GO:0110165), “Intracellular” and “Protein-containing complex” (GO:0032991) were annotated from the Cellular Component. Finally, “Binding”, “Catalytic activity” and “Transporter activity” (GO:0005215) were annotated from the Molecular Function category (**Figure 2**). In the KOG functional annotation, the three most annotated categories were “Posttranslational modification, protein turnover, chaperones”, “General function prediction only” and “Translation, ribosomal structure and biogenesis” (Online Resource 5: **Supplementary Figure S2**). Finally, in the KEGG pathway functional annotation, the pathways with the highest percentage of annotated genes were “Translation”, “Signal transduction” and “Carbohydrate metabolism” (**Figure 3**). The functional annotation analysis revealed that a total of 39,947 out of 94,058 annotated Unigenes belonged to the grape fungal community, as characterized by Iorizzo et al. (2023). These Unigenes were removed, and the subsequent analyses were conducted on 57,495 Unigenes.

**Table 3:** RNAseq assembly statistics.

Parameter	Value
Raw data	608 million
Clean reads	598 million
Q30%	91.54
GC content %	46.62
N° transcripts	226,498
N° unigenes	97,442
Transcripts N50 bp	2,753
Unigenes N50 bp	1,881
Mapping rate %	77.3
BUSCO Complete(single) Transcripts %	63.1
BUSCO Complete(single) Unigenes %	63.1

**Table 4:** Functional annotation statistics.

Database	Number of Unigenes	Percentage (%)
Annotated in NR	64,056	65.73
Annotated in NT	87,354	89.64
Annotated in KO	21,062	21.61
Annotated in SWISS-PROT	43,879	45.03
Annotated in PFAM	40,802	41.87
Annotated in GO	40,799	41.87
Annotated in KOG	18,034	18.5
Annotated in all databases	9,981	10.24
Annotated in at least one database	94,058	96.52
Total Unigenes	97,442	100

### 3.4 Sample clustering

**Figure 4:**
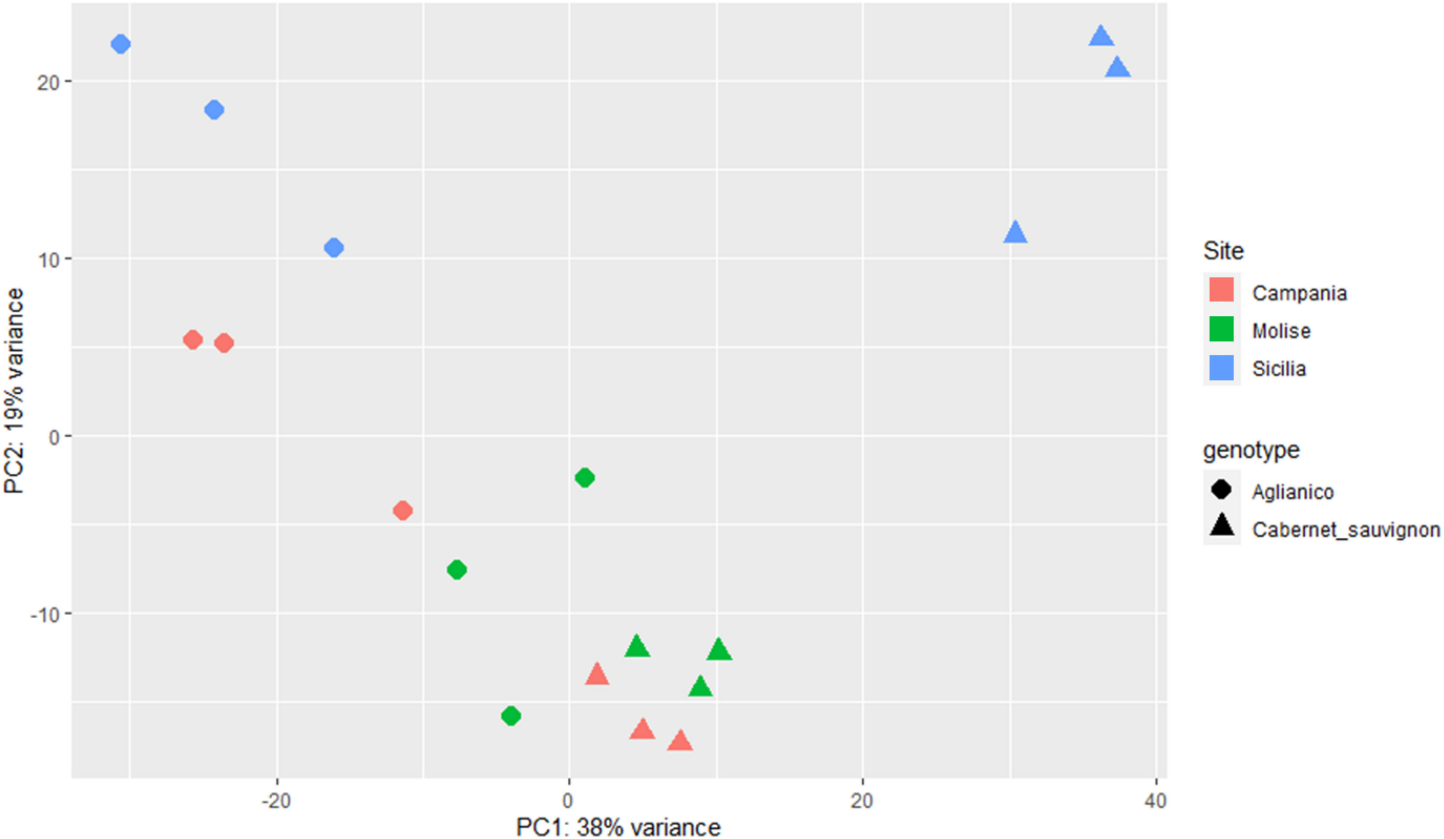
PCA of the six samples (A_CAM, A_MOL, A_SIC, C_CAM, C_MOL and C_SIC) using the normalized read counts as input data. The label shapes correspond to the genotypes (Aglianico, circle; Cabernet Sauvignon, triangle), and the colors correspond to the sites (see legend). Three biological replicates for each sample are reported.

With the aim of deciphering the transcriptomic profile of each sample, the Unigene read counts of the two genotypes cultivated at the three sites were used as inputs for PCA and explained 57% of the total variability (**Figure 4**). PCA revealed a sharp separation of Aglianico (circles) from Cabernet Sauvignon (triangles), suggesting that the differences in the transcriptomic profiles are due mainly to the genotype (**Figure 4**). Moreover, the green and red triangles grouped at the bottom of the figure indicate that Cabernet Sauvignon is less influenced by the growing site (CAM and MOL) than the Aglianico samples are, which shows a greater variance in PC2. However, the *weather* variable at the SIC site greatly influences the plant transcriptomic profiles of both genotypes (light blue circles and triangles) (**Figure 4**).

### 3.5 Identification of DEGs

**Figure 5:**
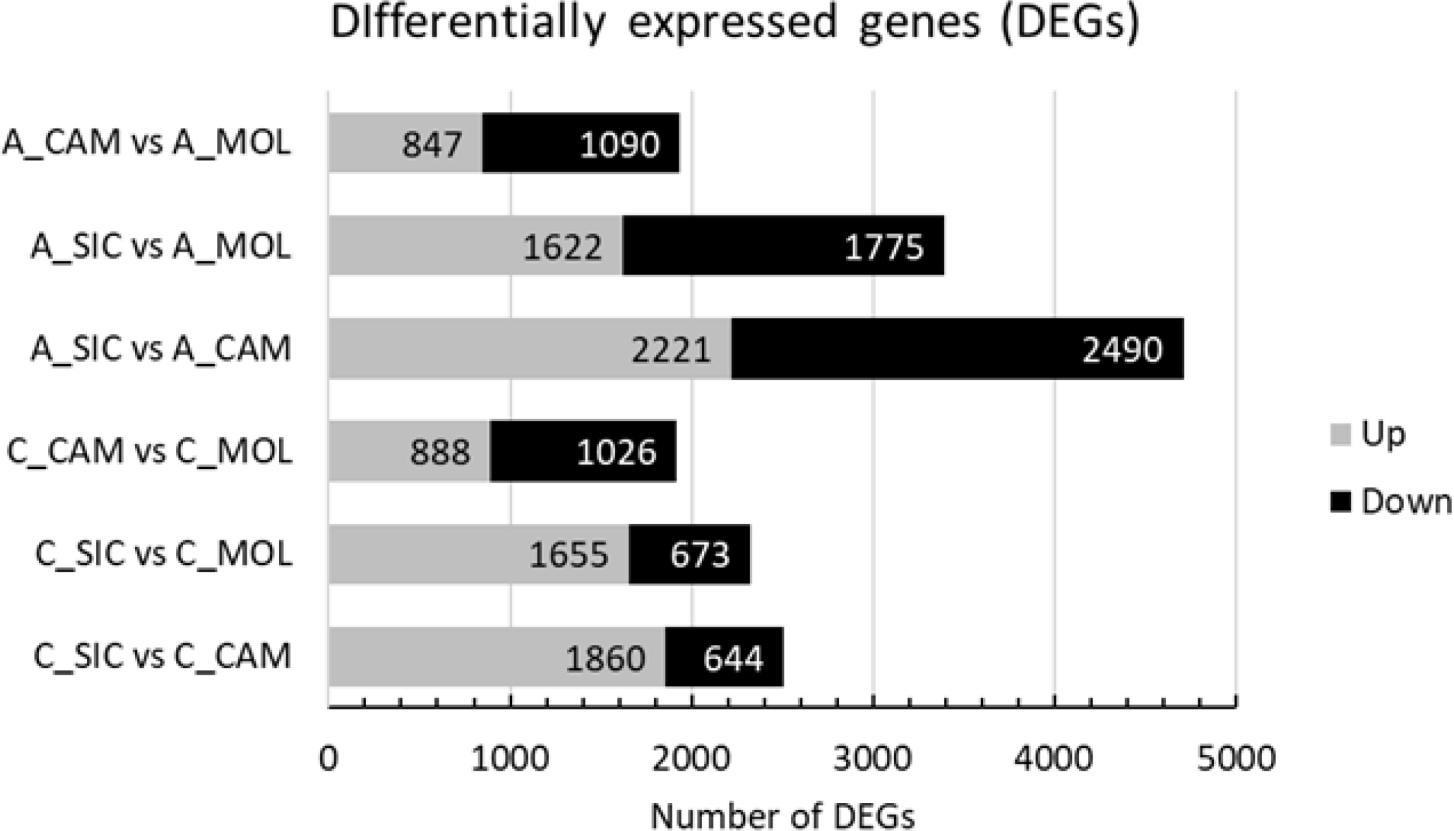
Number of differentially expressed genes (DEGs) in each comparison. A, Aglianico; C, Cabernet Sauvignon; CAM, Campania; MOL, Molise; SIC, Sicily.

The characterization of the Aglianico and Cabernet Sauvignon transcriptomes was accomplished by identifying those Unigenes with expression levels that changed as a function of the growing site. According to the experimental design, a total of 11,887 DEGs among the two genotypes and the three sites were identified, 1937 of which were differentially expressed in A_CAM vs. A_MOL (847 upregulated and 1090 downregulated), 3397 in A_SIC vs. A_MOL (1622 upregulated and 1775 downregulated), 4711 in A_SIC vs. A_CAM (2221 upregulated and 2490 downregulated), 1914 in C_CAM vs. C_MOL (888 upregulated and 1026 downregulated), 2328 in C_SIC vs. C_MOL (1655 upregulated and 673 downregulated) and 2504 in C_SIC vs. C_CAM (1860 upregulated and 644 downregulated) (**Figure 5**). The greatest number of DEGs was found in the Aglianico genotype (**Figure 5**). Furthermore, the comparisons that included the SIC site presented the greatest number of DEGs for both genotypes (**Figure 5**). Validation of the RNAseq experiment was performed by measuring the expression levels of ten selected DEGs by quantitative real-time PCR (qRT−PCR) (Online Resource 3: **Supplementary Table S3** and Online Resource 6: **Supplementary Figure S3**). The results revealed high congruence between the RNAseq and qRT−PCR results (coefficient of determination R^2^ = 0.93), which confirms the high reliability of the RNAseq analysis in the quantification of gene expression.

### 3.6 DEG functional enrichment

**Figure 6:**
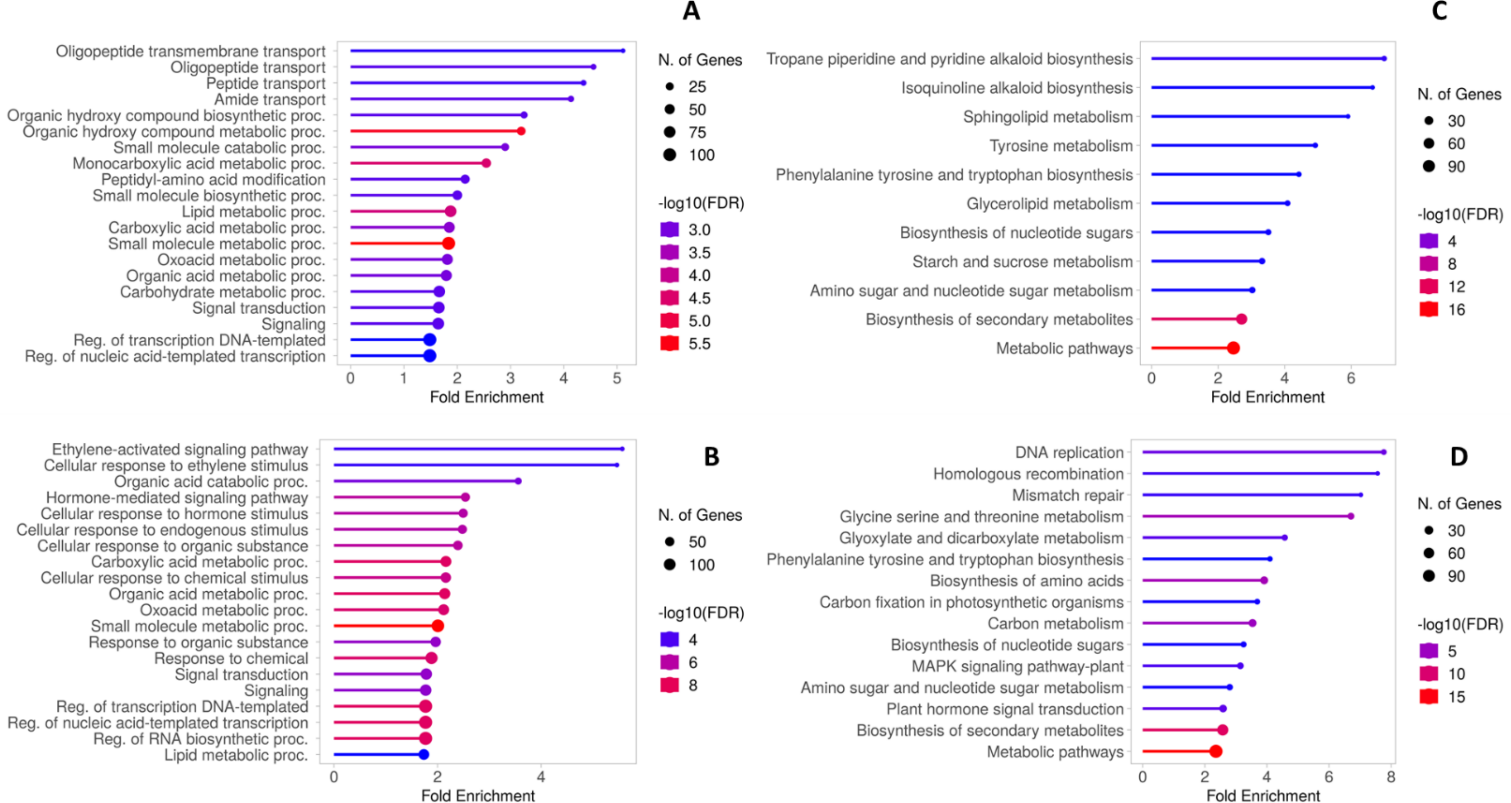
**(A)** Gene Ontology biological process enrichment of AGL DEGs. **(B)** Gene Ontology biological process enrichment of CAB DEGs. **(C)** KEGG functional enrichment of AGL DEGs **(D)** KEGG functional enrichment of CAB DEGs.

The enrichment of AGL and CAB DEGs in the GO and KEGG databases was performed to identify the principal pathways and biological processes involved in the transcriptome reprogramming of each genotype. Among the AGL enriched GO terms of the Biological Process, “Oligopeptide transmembrane transport”, “Amide transport” and “Organic hydroxy compound metabolic process” were the three most represented categories (**Figure 6A**). Differently, “Ethylene-activated signaling pathway”, “Organic acid catabolic process” and “Hormone-mediated signaling pathway” were the three most represented categories in CAB (**Figure 6B**). The main KEGG pathways in AGL were associated with the “Tropane piperidine and pyridine alkaloid biosynthesis” and “Isoquinolone alkaloid biosynthesis” categories, followed by “Sphingolipid metabolism” and “Tyrosine metabolism” (**Figure 6C**). As regards the KEGG pathways in CAB, “DNA replication”, “Homologous recombination” and “Mismatch repair” are the most represented, followed by “Glycine serine and threonine metabolism” and “Phenylalanine tyrosine and tryptophan biosynthesis” (**Figure 6D**).

### 3.7 Genotype and site specificity of gene expression

The transcriptomic response of each genotype at the three distinct sites was evaluated by recruiting clusters that were expressed in a single genotype per site (FPKM ≠ 0) and were completely unexpressed at the other sites (FPKM = 0). In particular, a total of 185 genes were specifically expressed in A_CAM, 90 genes in A_MOL, 171 genes in A_SIC, 226 genes in C_CAM, 165 genes in C_MOL, and 206 genes in C_SIC, all of which represent a specific transcriptomic signature of the genotype at that particular site. Most of these genes were not annotated and encoded hypothetical and/or uncharacterized proteins (data not shown). In **Table 5**, some genes of interest are listed along with their cluster ID, annotation, accession number, e-values and specific site. In A_CAM, probable genes encoding serine/threonine-protein kinase WNK5 (cluster-39.0) (Su et al., 2024) and DExH-box ATP-dependent RNA helicase (cluster-27627.0) involved in ribosome biogenesis (Liu and Imai, 2018) were specifically expressed. The gene encoding vegetative cell wall protein gp1 (cluster 27903.60085), which functions as a major component of the outer cell wall layer (Niu et al., 2020), was among the genes specifically expressed in A_MOL, along with the gene encoding UDP-glycosyltransferase 85A2-like (cluster-1161.0), which is involved in the key step of secologanin biosynthesis (Jin et al., 2022). The genes encoding phenylalanine N-monooxygenase (cluster-27903.31192), which is part of the pathway that converts phenylalanine to glucosinolate; glucotropaeolin (Wittstock and Halkier, 2000); and myb-related protein 315 (cluster-27903.28077), which confers cold and drought tolerance (Fang et al., 2024), were exclusively expressed in A_SIC. Clusters specific to C_CAM encode proteins homologous to the homeobox-leucine zipper protein ATHB-15 (cluster-27903.3260) (Li et al., 2020) and an endo-1,4-beta-mannosidase 5-like (cluster-27903.633) (Rahmani et al., 2017). The transcription factor JUNGBRUNNEN 1 (cluster-17927.0), a regulator of drought tolerance in tomatoes (Thirumalaikumar et al., 2018), and MLP-like protein 43 (cluster-27903.61696), which confers drought tolerance (Fujita et al., 2021), are among the genes specifically expressed in C_MOL. Finally, the specific expression of an APETALA2/ethylene responsive factor (cluster-20558.0), which is involved in berry firmness (Wong et al., 2016), and an SRG1 protein (cluster-21223.0) (Cui et al., 2018) were detected in C_SIC.

**Table 5:** Genotype and site specificity of gene expression.

Cluster ID	Annotation v4.1	PN40024.v4.1_REF_prot	e-value	Site	Reference
39.0	probable serine/threonine-protein kinase WNK5 (XP_019077023.1)	Vitvi08g00843_t001	5.50×10^-11^	A_CAM	Su et al., 2024
27627.0	DExH-box ATP-dependent RNA helicase DExH3 isoform X1 (XP_002269787.1)	Vitvi18g01725_t001	5.94×10^-105^	A_CAM	Liu and Imai, 2018
27903.60085	vegetative cell wall protein gp1 (XP_010648854.2)	Vitvi04g01979_t001	1.58×10^-41^	A_MOL	Niu et al., 2020
1161.0	UDP-glycosyltransferase 85A2-like (NP_001277170.1)	Vitvi18g00421_t001	1.10×10^-62^	A_MOL	Jin et al., 2022
27903.31192	Phenylalanine N-monooxygenase (RVX19945.1)	Vitvi06g01205_t001	2.58×10^-29^	A_SIC	Wittstock and Halkier, 2000
27903.28077	myb-related protein 315 (XP_010662108.1)	Vitvi16g01449_t001	0	A_SIC	Fang et al., 2024
27903.3260	homeobox-leucine zipper protein ATHB-15 (XP_002284003.2)	Vitvi09g00310_t001	8.03×10^-11^	C_CAM	Li et al., 2020
27903.633	mannan endo-1,4-beta-mannosidase 5-like (XP_034674580.1)	Vitvi18g02343_t001	0.0	C_CAM	Rahmani et al., 2017.
17927.0	transcription factor JUNGBRUNNEN 1 (XP_002265591.3)	Vitvi19g01566_t001	3.60×10^-63^	C_MOL	Thirumalaikumar et al., 2018
27903.61696	MLP-like protein 43 (XP_034672643.1)	Vitvi01g01977_t001	9.23×10^-109^	C_MOL	Fujita et al., 2021
20558.0	ethylene-responsive transcription factor 2 (XP_003633905.1)	Vitvi15g01202_t001	3.09×10^-28^	C_SIC	Wong et al., 2016
21223.0	protein SRG1 (XP_002269890.1)	Vitvi10g00687_t001	2.71×10^-55^	C_SIC	Cui et al., 2018.

### 3.8 WGCNA

**Figure 7:**
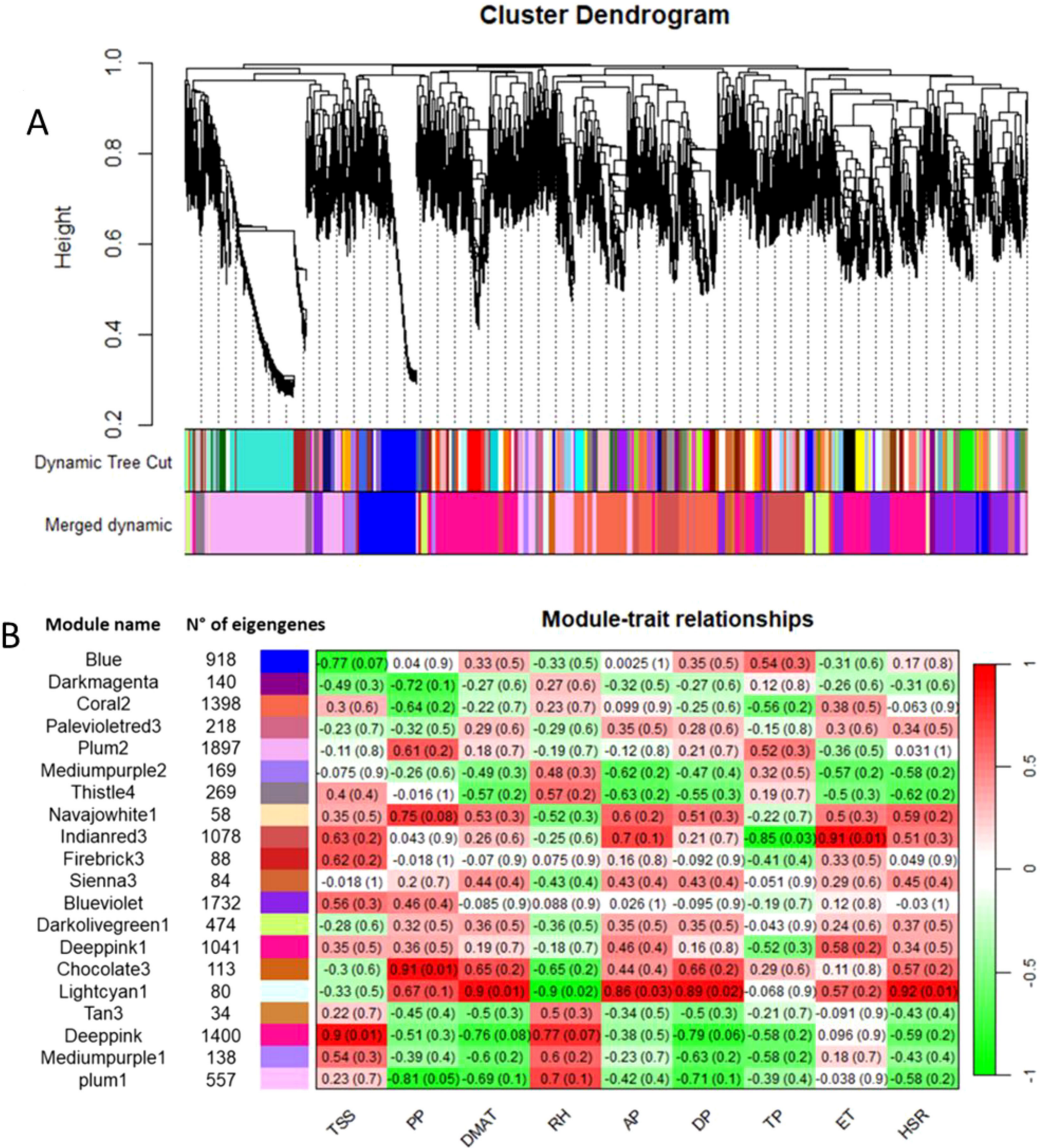
**(A)** Clustering dendrogram of genes, with dissimilarity based on topological overlap, together with assigned module colors. **(B)** Number of eigengenes in each module and heatmap showing the Pearson correlation among the eigengenes in co-expressed gene modules. The total soluble solids (TSS), polyphenols (PP), daily maximum air temperature (DMAT), relative humidity (RH), air pressure (AP), dew point (DP), total precipitation (TP), evapotranspiration (ET) and hours of sun radiation (HSR) were measured.

Co-expression networks of weighted genes associated with the weather course parameters and quality traits (DMAT, RH, AP, DP, TP, ET, and HSR values as weather course parameters; TA, TSS, ANTH, MI and PP values as quality traits) of each site were constructed by WGCNA based on 11,887 DEGs. These genes were grouped into 20 co-expressed modules (**Figure 7A, B**). Each set of highly correlated genes corresponded to a branch of the tree (**Figure 7A**). With respect to the weather course parameters, DMAT (positive correlation, r^2^ = 0.9), RH (negative correlation, r^2^ = -0.9), AP (positive correlation, r^2^ = 0.86), DP (positive correlation, r^2^ = 0.89) and HSR (positive correlation, r^2^ = 0.92) were significantly correlated with the “lightcyan1” module (80 genes), and both TP (negative correlation, r^2^ = -0.85) and ET (positive correlation, r^2^ = 0.91) were significantly correlated with the “indianred3” module (1078 genes) (**Figure 7B**). With respect to the quality traits, ANTH, TA and MI were not significantly correlated with any module (data not shown). Conversely, co-expression modules that were highly correlated with TSS and PP were identified; in particular, the “deeppink” module (1400 genes) was significantly correlated with TSS (positive correlation, r^2^ = 0.9), and two modules, namely, “chocolate3” (113 genes) (positive correlation, r^2^ = 0.91) and “plum1” (557 genes) (negative correlation, r^2^ = -0.81), were significantly correlated with PP (**Figure 7B**). The genes in these latter modules were filtered according to the highest intramodular connectivity (hub genes, module membership [MM] > 0.65 and gene significance [GS] > 0.65), as these genes might represent points of biological interest in defining plant adaptability to the environment (Online Resource 7: **Supplementary Figure S4**).

### 3.9 Biological function analysis of the hub genes

**Figure 8:**
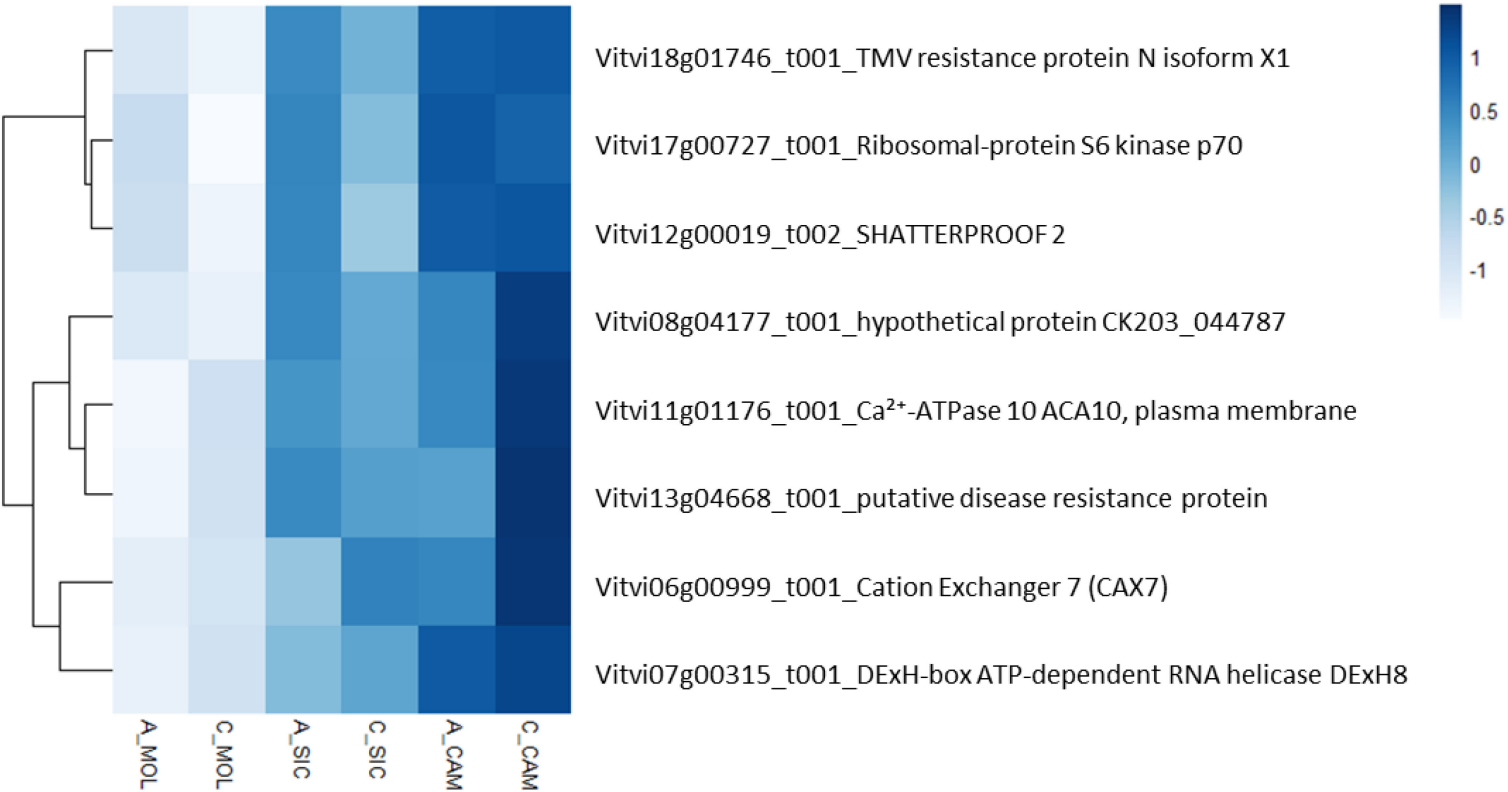
Heatmap representing the relative expression of the eight genes in the lightcyan1 module that correlated with the DMAT, RH, AP, DP and HSR traits at all the sites under investigation.

To describe the biological functions of the hub genes, the functional categories annotated in the PN40024 v4 *Vitis vinifera* genome were considered. Although the modules included a substantial number of genes, most of them encode unknown or uncharacterized proteins, resulting in very few enriched genes in each functional category. As shown in **Table 6**, TSS was positively correlated with the deeppink module, with a total of 326 genes involved in several functional categories, such as cellular component organization and biogenesis (10 genes), nucleic acid metabolism (18 genes), protein metabolism and modification (14 genes), regulation of transcription (31 genes), hormone signaling (14 genes), signaling pathways (12 genes), and transport (19 genes). PP was positively correlated with the chocolate3 module (32 genes), containing genes involved in cellular component organization and biogenesis (3 genes), amino acid metabolism (4 genes), regulation of transcription (6 genes), and signaling pathways (2 genes). Interestingly, PP was also negatively correlated with the plum1 module (121 genes), including genes involved in amino acid metabolism (6 genes), protein metabolism and modification (9 genes), phenylpropanoid metabolism (3 genes), terpenoid metabolism (3 genes), regulation of transcription (9 genes), hormone signaling (5 genes), and transport (10 genes). A substantial number of genes are included in the indianred3 module (255 genes), which is negatively correlated with TP; most of the genes are involved in cytoskeleton organization and biogenesis (2 genes), lipid metabolism (3 genes), and the regulation of transcription (4 genes) (**Table 6** and Online Resource 8: **Supplementary Table S4**). ET was positively correlated with the indianred3 model (347 genes), with genes involved in lipid metabolism (3 genes), protein metabolism and modification (3 genes), and signaling (6 genes) (**Table 6** and Online Resource 8: **Supplementary Table S4**). As previously described, most of the weather course traits were significantly correlated with the lightcyan1 module (DMAT, RH, AP, DP and HSR with 18, 16, 21, 15 and 31 genes, respectively) (**Table 6** and Online Resource 8: **Supplementary Table S4**). These genes are related mainly to the regulation of transcription (4 genes), signaling (4 genes) and transport (5 genes). Eight genes are shared among the weather course traits: a cation transporter (autoinhibited Ca²^+^-ATPase 10, ACA10), a plasma membrane protein [Vitvi11g01176]; a cation exchanger (cation exchanger 7, CAX7) [Vitvi06g00999_t001]; SHATTERPROOF 2 [Vitvi12g00019_t002]; a transcription factor (DExH-box ATP-dependent RNA helicase, DExH8 [Vitvi07g00315_t001]); a disease resistance protein (PREDICTED: TMV resistance protein N isoform X1 [Vitvi18g01746_t001], putative disease resistance protein [Vitvi13g04668_t001]); and a serine/threonine kinase (Ribosomal-protein S6 kinase p70 [Vitvi17g00727_t001]). As shown in **Figure 8**, the expression levels of these genes in both genotypes and at all three sites increased with increasing HSR and DMAT and decreasing RH, indicating a decreasing expression pattern from the Campania site to the Molise site.

**Table 6:** Module\trait correlation.

Module\Trait	Eigengenes (GS and MM > 0.65)	Trait trend	Correlation	Main functional category
Deeppink\TSS	326	SIC<CAM<MOL	Positive	cellular component organization and biogenesis, nucleic acid metabolism, protein metabolism and modification, regulation of transcription, hormone signaling, signaling pathway, transport
Chocolate3\PP	52	MOL<CAM<SIC	Positive	cellular component organization and biogenesis, amino acid metabolism, regulation of transcription, signaling pathway
Plum1\PP	121	MOL<CAM<SIC	Negative	Amino acid metabolism, protein metabolism and modification, phenylpropanoid metabolism, terpenoid metabolism, regulation of transcription, hormone signaling, transport
Lightcyan1\DMAT	18	MOL<SIC<CAM	Positive	Regulation of transcription, signaling, transport
Lightcyan1\RH	16	CAM<SIC<MOL	Negative	Regulation of transcription, signaling, transport
Lightcyan1\AP	21	MOL<SIC<CAM	Positive	Regulation of transcription, signaling, transport
Lightcyan1\DP	15	MOL<SIC<CAM	Positive	Regulation of transcription, signaling, transport
Lightcyan1\HSR	31	MOL<SIC<CAM	Positive	Regulation of transcription, signaling, transport
Indianred3\TP	255	CAM<MOL<SIC	Negative	Cytoskeleton organization and biogenesis, lipid metabolism, regulation of transcription
Indianred3\ET	347	SIC<MOL<CAM	Positive	Lipid metabolism, protein metabolism and modification, signaling

For each module/trait, the number of included genes, the trait trend (from the site with the lowest value to the site with the highest value), the type of correlation between the trait value and eigengene expression, and the main functional categories are reported.

### 3.10 Hub transcription factors

**Figure 9:**
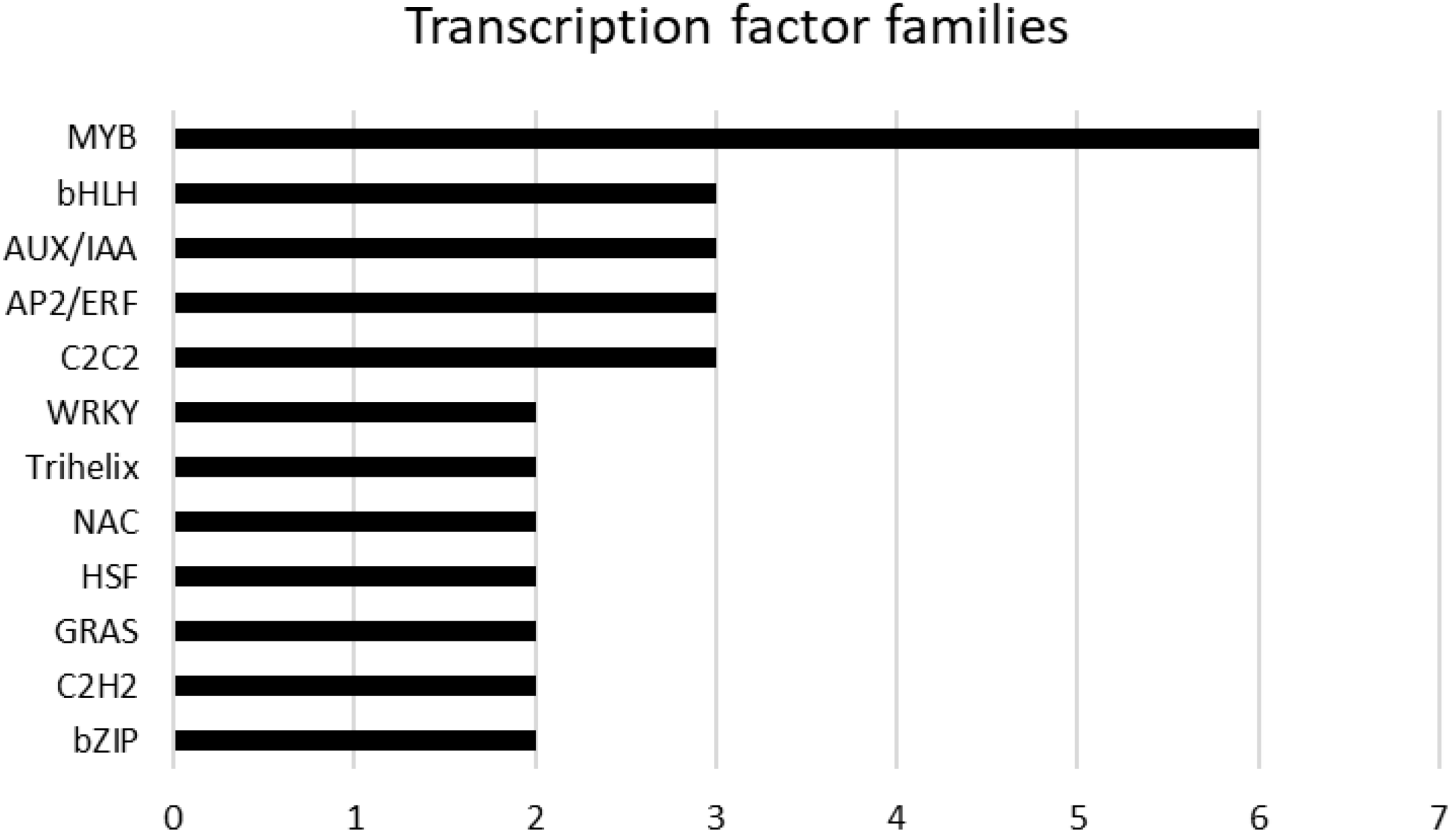
Hub genes belonging to transcription factor families. The families are sorted according to the number of genes.

A total of 58 genes encoding transcription factors were retrieved since all the modules were significantly correlated with a trait (Online Resource 9: **Supplementary Table S5**). Most of these genes belong to the MYB, bHLH, AUX/IAA, AP2/ERF, C2C2 and WRKY families (**Figure 9**). **Table 7** lists the genes whose expression greatly changed among sites. Specifically, MYB61 regulates a specific set of target genes by binding DNA to the AC within the cis-element 5’-ACCTAC-3’. Higher expression of this gene was observed in both genotypes grown at the CAM site, which had higher ET and HSR values and lower TP values. Cluster-27903.36947 encodes multiprotein-bridging factor 1C (MBF1C), a transcriptional coactivator that stimulates transcriptional activity by bridging regulatory proteins and TBP, recruiting TBP to promoters occupied by DNA-binding regulators. This gene was more highly expressed in Aglianico than in Cabernet Sauvignon at all sites. Cluster-27903.38074 encodes *Arabidopsis* pseudo-response regulator 7 (APRR7) involved in the positive and negative feedback loops of the circadian clock. This gene was highly expressed at the MOL site, where both the DMAT and HSR were lower than those at the other sites. Cluster-27903.17293 encodes a CPC (CAPRICE) MYB transcription factor that determines the fate of epidermal cell differentiation. This gene was more highly expressed in Aglianico than in Cabernet Sauvignon at sites with low TP (Campania and Molise).

**Table 7:** Transcription factors highly influenced by site and/or genotype.

Cluster ID	PN40024.v4.1_REF_prot	A_SIC	A_MOL	A_CAM	C_SIC	C_MOL	C_CAM	Functional annotation
27903.36947	Vitvi11g00351_t001	41.91	193.72	53.03	22.68	30.45	20.55	Multiprotein-bridging factor 1c MBF1C (Q9LV58)
27903.22182	Vitvi15g04655_t001	1.07	1.09	3.29	1.8	1.42	2.23	Transcription factor MYB61 (Q8VZQ2)
27903.17293	Vitvi10g01641_t001	1.10	6.47	2.25	0.18	0.97	0.36	Myb caprice CPC (O22059)
27903.38074	Vitvi06g00368_t001	4.37	12.10	6.59	5.07	9.1	5.34	Pseudoresponse regulator 7 (APRR7) (Q93WK5)

The PN40024.v4.1_REF_prot column shows the *Vitis vinifera* genome v4 annotation. Gene expression is reported as FPKM.

## 4 Discussion

Temperature, water, light, and CO_2_ concentration are among the most important environmental factors that affect vine and fruit development by interacting with the genotype (Rienth et al., 2021). These factors are expected to be largely modulated by global climate change. The predicted and already observed consequences of climate change on wine quality include higher alcohol content, lower acidity, and altered aroma profiles (Schultz, 2016; Pons et al., 2017b; van Leeuwen and Destrac-Irvine, 2017), leading to a loss of typicity and terroir expression. Fortunately, vine cultivars have shown adaptability to different mesoclimates (Morales-Castilla et al., 2020), making grapevines an interesting model for studying the genetic and molecular bases that underlie phenotypic plasticity (Dal Santo et al., 2013, 2016, 2018). The analysis of genotype-based transcriptional modifications influenced by GxE interaction is crucial for understanding the different regulatory mechanisms of metabolic pathways during berry ripening (Ferrandino et al., 2023; Dal Santo et al., 2013; Massonnet et al., 2017). However, a precise definition of the environment component (E) is often unattainable in open-field studies. In this study, we investigated the GxE interactions of two different red grape varieties grown across three different environments in central-southern Italy by combining and correlating transcriptomic data with weather indices and berry quality traits. Our goals were to overcome the challenge of a precise definition of the E component in GxE studies and to minimize the differences in berry phenology at each site at harvest time. We defined the concept of the *weatherome*, a combination of weather course indices used to characterize the three sites during the period surrounding the harvest. This weather characterization showed that Campania was the hottest and sunniest site, with a ET value and low values of both RH and TP during the thirty days before harvest. Conversely, Molise was the coldest and cloudiest site, with a high value of RH. Finally, Sicily was the rainiest site and was characterized by low ET values. These climatic conditions led to the lowest TSS content occurring in Sicily (Nicolosi et al., 2022), which was probably due to the greater amount of water in the environment diluting the berries’ juice. Increased temperatures during ripening have been shown to lead to altered compositions of secondary metabolites, such as phenolic and aroma-conferring compounds, in grapes (van Leeuwen and Destrac-Irvine, 2017; Abeysinghe et al., 2019). The data collected for the Cabernet Sauvignon genotype corroborate these findings, as the levels of phenolics were increased on average in both Campania and Sicily, where high DMAT values have been reported. Moreover, Cabernet Sauvignon intrinsically presented higher MI values than did Aglianico at all the sites. The transcriptome analysis highlighted the influence of the environment on genotype adaptation and behavior. In fact, different tendencies in transcriptomic remodeling of the two genotypes in response to the growing environment were observed. Compared with Aglianico, Cabernet Sauvignon presented lower transcriptome reprogramming across the sites. This result confirms what was reported by Dal Santo et al. (2018). According to their results, the Cabernet Sauvignon transcriptome remained more stable across vintages and locations, this contributing to the success of this cultivar in many different parts of the world (Dal Santo et al., 2018). In contrast, Aglianico markedly perceives the changing environment and adapts to it by modifying the regulation of gene expression. This result is consistent with what was reported by Nicolosi et al. (2022), who analyzed berry morphophysiological characteristics and found high phenotypic plasticity in Aglianico, whereas Cabernet Sauvignon presented increased potential in terms of bud performance, shoot growth, leaf area, and total leaf area/vine (Nicolosi et al., 2022). Furthermore, the different transcriptomic reprogramming observed between the two genotypes was also qualitative. The Gene Ontology biological process enrichment clearly indicates that Aglianico responds to the changing environment mainly with the deregulation of “Peptide” and “Solute transport” categories. On the other hand, in Cabernet Sauvignon a significant enrichment of DEGs involved in the ethylene-dependent signaling pathways was registered, probably explaining the higher maturity index of Cabernet Sauvignon berries than Aglianico. However, the aforementioned divergent ability of the genotypes to respond to changing environments is minimal in Sicily, where both Aglianico and Cabernet Sauvignon underwent strong transcriptome reprogramming. This results might indicate that in Sicily the weather parameters reached values globally representing the limit beyond which also Cabernet Sauvignon had to deregulate gene expression.

The analysis of the transcriptomic data also led to the identification of a specific gene list characterizing the gene expression at a specific site. Notably, in A_MOL, the specific expression of the gene encoding 7-deoxyloganetin glucosyltransferase-like, which is involved in the key step in secologanin biosynthesis (Jin et al., 2022), was detected. Secologanin is a secoiridoid monoterpene synthesized from geranyl pyrophosphate through the mevalonate pathway. Smoke exposure has been shown to increase the activity of 7-deoxyloganeticacid glucosyltransferase in grapevines to facilitate the production of iridoids and defend the plant against the reactive oxygen species present in smoke (Szeto et al., 2024). Therefore, we can hypothesize that Molise experienced some bush fires and that Aglianico was more sensitive to smoke than Cabernet Sauvignon. Phenylalanine N-monooxygenase (cluster-27903.31192), which is part of the pathway that converts phenylalanine to glucotropaeolin, was exclusively expressed in A_SIC, where it might contribute to the characteristic flavor of the wine in a site-specific manner. Notably, this gene was not expressed in Aglianico grown in Campania even during a previous trial (Villano et al., 2023). In addition, the overall weather conditions in Molise suggested that a water deficit did not occur. However, the transcription factors JUNGBRUNNEN 1 and MLP-like protein 43, both of which are involved in abiotic stress tolerance, were among the genotype/site-specific genes expressed in Cabernet Sauvignon, indicating the high basal strength of this genotype in facing abiotic stress compared to Aglianico at that specific site. The analysis of the relationships among co-expression modules (deeppink, chocolate3, plum1 and indianred3) and weather and quality traits suggested that the traits correlated with the expression of a limited group of genes involved mainly in signaling, transcription regulation and transport, thus indicating the crucial role of these biological processes in establishing the GxE interaction. The relationships between the lightcyan1 co-expression module and weather factors, such as DMAT, RH, AP, DP, and HSR, suggested a significant correlation with the expression of a restricted group of genes that are involved mainly in transport mechanisms. Two of these genes encode cation transporters, namely ACA10, which is known to be involved in sequestering free cytosolic Ca^2+^ to the endoplasmic reticulum (Sunitha et al., 2019; Geisler et al., 2000), and CAX7, a vacuolar membrane-localized K^+^-dependent Na^+^/Ca^2+^ transporter, that exports cations of the cytosol to maintain optimal ionic concentrations in the cell (Shigaki et al., 2006). Both of them are upregulated under both high-temperature and high-sun radiation conditions and downregulated under high-RH conditions. These results suggest that an eventual adaptive mechanism towards the growth environment might involve ion movement across cell compartments, and more specifically, the removal of cytosolic Ca^2+^ ions. Cabernet Sauvignon had the highest expression level of these transporters, suggesting that the mechanisms previously described might play a pivotal role in the high adaptability of Cabernet Sauvignon to different growing environments. Furthermore, the regulation of ACA10 expression has been shown to be indirectly related to ANTH accumulation. Frohnmeyer et al. (1999) demonstrated that an increase in free cytosolic Ca^2+^ levels is associated with the induction of chalcone synthase (CHS) expression, namely, the gene involved in the first committed step of ANTH biosynthesis. Cytosolic Ca^2+^ accumulation is a consequence of the downregulation of ACA10 mediated by miR5225, miR3627, and miR4376, indirectly promoting both the induction of CHS expression and an increase in ANTH content (Sunitha et al., 2019). This mechanism is strongly consistent with our results since both Aglianico and Cabernet Sauvignon samples grown in Campania with a relatively high expression of ACA10 also presented low ANTH contents. However, CHS transcripts were not identified among the significant DEGs (log_2_FoldChange padj > 0.05). Furthermore, in our previous work, reduced ANTH accumulation along with low or insignificant levels of biosynthesis-related gene expression, including CHS, were reported at sites where high temperatures and low thermal excursions were registered (Iorizzo et al., 2023).

Finally, some hub genes correlated with weather course and quality traits are important transcription factors. Among them, MYB61 is a transcriptional regulator of stomatal closure and regulates stomatal pore size in an abscisic acid-independent manner (Liang et al., 2005). Greater expression of this gene was recorded in the two genotypes grown in Campania, the site with the lowest TP and the highest ET and HSR, suggesting that both genotypes counteract low water availability by regulating stomatal function. The transcription factor MBF1C is involved in tolerance to heat and osmotic stress through the partial activation of the ethylene-response signal transduction pathway (Tsuda et al., 2004; Suzuki et al., 2005, 2008). Interestingly, MBF1C was more highly expressed in the Aglianico genotype than in the Cabernet Sauvignon genotype at all the sites. This result highlights the lower adaptability of Aglianico to different environments since it needs to activate tolerance mechanisms in response to changing environmental cues. APRR7 represses the expression of clock proteins and master regulators of plant growth, development, and response to abiotic stress (Kaczorowski and Quail, 2003; Nakamichi et al., 2010; Salomé et al., 2010; Liu et al., 2013). APRR7 was highly expressed in plants at the Molise site, where both the DMAT and HSR were lower than those at the other sites. Additionally, the CPC MYB transcription factor represses trichome development through lateral inhibition. It has been suggested that, together with GL3 (GLABRA3) or basic-helix-loop-helix (bHLH) transcription factors, CPC MYB promotes the formation of developing hair cells (H position) in the root epidermis, probably by inhibiting non-hair cell formation (Lee and Schiefelbein, 2002; Schellmann et al., 2002). The increased expression levels in Aglianico at the sites with low TP (Campania and Molise) might indicate that Aglianico is more sensitive to a lack of water than Cabernet Sauvignon is. Aglianico might respond to this condition by inhibiting the lateral development of roots and promoting vertical development with the aim of exploring the soil to reach water.

## 5 Conclusions

In this study, transcriptome analysis was performed on two grapevine varieties grown at three different sites in central-southern Italy (Campania, Molise and Sicily). The data were combined with several weather course indices using WGCNA. Moreover, considering the main role of TSS and PP in wine quality, the correlation of these parameters with the transcriptomic profiles at each site was also determined. From a climatic point of view, Campania was the hottest and sunniest site and was also characterized by low RH and TP values. Conversely, Molise was the coldest and cloudiest site, with high RH, whereas Sicily was the rainiest site. With respect to the transcriptomic response to different environmental conditions, a sharp difference in transcriptomic plasticity between the two genotypes was observed, as Cabernet Sauvignon presented less transcriptome remodeling than Aglianico did, however, in Sicily, both Aglianico and Cabernet Sauvignon underwent strong transcriptome reprogramming. WGCNA suggested that most of the weather course parameters were correlated with the expression of a limited group of genes involved mainly in signaling and transport mechanisms. We propose a model in which low levels of ACA10 cation transporter expression might be responsible for CHS induction. Consistently, samples of both Aglianico and Cabernet Sauvignon grown in Campania with higher expression of ACA10 also presented low ANTH contents. Hub transcription factors correlated with weather course and quality traits were identified and are likely involved in crucial pathways and processes such as stomatal closure and stomatal pore size (MYB61), tolerance to heat and osmotic stress (MBF1C), regulation of plant growth (APRR7), and inhibition of lateral root development (CPC). Finally, the genotype/site specificity of gene expression led to the identification of a transcriptomic profile that may specifically identify the genotype and its growing site.

## Data Availability

The datasets presented in this study can be found in online repositories. The names of the repository/repositories and accession number(s) can be found in the article/**Supplementary Material**.
