# Supplementary material for: Study of red vine phenotypic plasticity across central-southern Italy sites: an integrated analysis of the transcriptome and weather indices through WGCNA
Source: Front Plant Sci. 2024 Nov 11;15:1498649. doi: 10.3389/fpls.2024.1498649 (PMC11586177; doi:10.3389/fpls.2024.1498649)
Supplement: Supplementary file 1 [file DataSheet1.zip › Online resource 1.pdf]

Online resource 1: Table S1. Geolocations and elevation of sites.

Site	Latitude	Longitude	Elevation [m]
Molise (San Biase, CB)	41°72’ N	14°57’ E	600
Campania (Galluccio, CE)	41°33’ N	13°89’ E	125
Sicilia (Zafferana Etnea, CT)	37°41’ N	15°7’ E	720
