# Supplementary material for: Study of red vine phenotypic plasticity across central-southern Italy sites: an integrated analysis of the transcriptome and weather indices through WGCNA
Source: Front Plant Sci. 2024 Nov 11;15:1498649. doi: 10.3389/fpls.2024.1498649 (PMC11586177; doi:10.3389/fpls.2024.1498649)
Supplement: Supplementary file 1 [file DataSheet1.zip › Online resource 2.pdf]

Online resource 2: Table S2. Number of raw and high-quality reads in each of the three biological replicates.

Sample	Raw_reads	Clean_reads
A_SIC1	30839659	30434310
A_SIC2	30903403	30094001
A_SIC3	34558109	33890291
C_SIC1	33110067	32582649
C_SIC2	36002031	35560210
C_SIC3	39750445	39084643
A_MOL1	30619062	30174766
A_MOL2	30799840	30196522
A_MOL3	30656260	30139376
C_MOL1	34668516	34230558
C_MOL2	30394310	29957152
C_MOL3	34236348	33854551
A_CAM1	41858490	41101642
A_CAM2	29821832	29286290
A_CAM3	37832323	37091607
C_CAM1	32683581	32063204
C_CAM2	32714385	32007643
C_CAM3	36602285	36064557
