# Supplementary material for: Study of red vine phenotypic plasticity across central-southern Italy sites: an integrated analysis of the transcriptome and weather indices through WGCNA
Source: Front Plant Sci. 2024 Nov 11;15:1498649. doi: 10.3389/fpls.2024.1498649 (PMC11586177; doi:10.3389/fpls.2024.1498649)
Supplement: Supplementary file 1 [file DataSheet1.zip › Online resource 3.pdf]

Online Resource 3: Table S3. Primer sequences and relative melting temperature used for real-time qPCR validation

Primer ID	Cluster ID	Sequence 5'->3'	Tm (°C)
Vv_cytP450_for<br>Vv_cytP450_rev	Cluster-27903.31320	CTCGTATCTGTCGTCGTGGT<br>AATAGCGGTCCATAGCGGAG	56
Vv_enChitA_for<br>Vv_enChitA_rev	Cluster-27903.46352	AATACCCCACTCCCAAGTCCA<br>AGGGCTGCCAAAAGGACTC	56
Vv_stSyn3_for<br>Vv_stSyn3_rev	Cluster-27903.58614	TAAACGCCATCGGAGAGTGG<br>ACAAGAACACGGGCACCTAC	56
Vv_flHydr_for<br>Vv_flHydr_rev	Cluster-27903.38163	GCGCGGGATGAAGCATTTAC<br>CGCTGAAGTATCTGTCCCCG	58
Vv_unchar_for<br>Vv_unchar_rev	Cluster-27903.25111	GTGGAGGCACTCAAGGATCAG<br>AGAGAGCTTCTTGGCCTGAG	58
Vv_MDIS1irlk_for<br>Vv_MDIS1irlk_rev	Cluster-27903.37365	AATACCTGAGCACTTGGGCG<br>AACATACGACAAGGCAGGCA	56
Vv_thauLike1_for<br>Vv_thauLike1_rev	Cluster-27903.32857	GTCGAAGGCAGCAGCAAATC<br>GATGGTGTAATCGGCTCCGT	56
Vv_prp10.8_for<br>Vv_prp10.8_rev	Cluster-27903.30237	ATGGAGGAGCTGGAACCATTG<br>GCTACCTCCATCTGTTGCCTC	58
Vv_STran3.1_for<br>Vv_STran3.1_rev	Cluster-27903.31642	GCGCCTCTAACGTCTGTGAT<br>ACCCGAAACCCAGTTCAGAC	56
Vv_prp1_for<br>Vv_prp1_rev	Cluster-27903.36056	CCGATGCAGTGAACATGTGG<br>GATAGTTGCCCGGTGGATCG	58
Vv_UBI60S_for<br>Vv_UBI60S_rev	Housekeeping (Cheng et al., 2021b)	TCTGAGGCTTCGTGGTGGTA<br>AGGCGTGCATAACATTTGCG	58
