# Supplementary material for: Study of red vine phenotypic plasticity across central-southern Italy sites: an integrated analysis of the transcriptome and weather indices through WGCNA
Source: Front Plant Sci. 2024 Nov 11;15:1498649. doi: 10.3389/fpls.2024.1498649 (PMC11586177; doi:10.3389/fpls.2024.1498649)
Supplement: Supplementary file 1 [file DataSheet1.zip › Online resource 7.pdf]

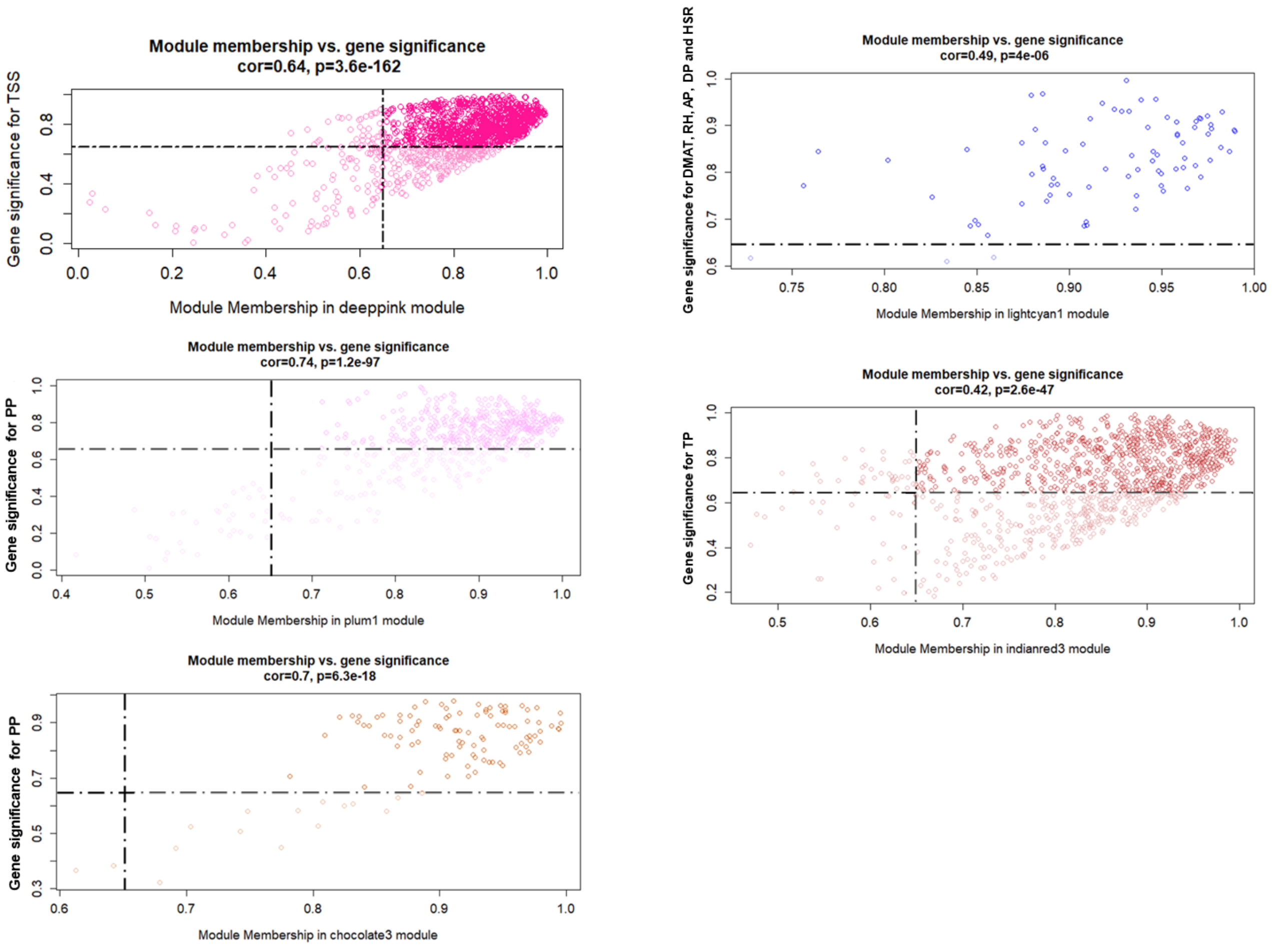

Online Resource 7: Figure S4. Gene Significance (GS) and Module Membership (MM) of the eigengenes belonging to the modules significantly correlated with both climatic indices and quality traits. X axis represents the MM in the module, Y axis represents the GS for the trait. A threshold of 0.65 is set.
