# Supplementary figures and images for: Study of red vine phenotypic plasticity across central-southern Italy sites: an integrated analysis of the transcriptome and weather indices through WGCNA
Source: Front Plant Sci. 2024 Nov 11;15:1498649. doi: 10.3389/fpls.2024.1498649 (PMC11586177; doi:10.3389/fpls.2024.1498649)

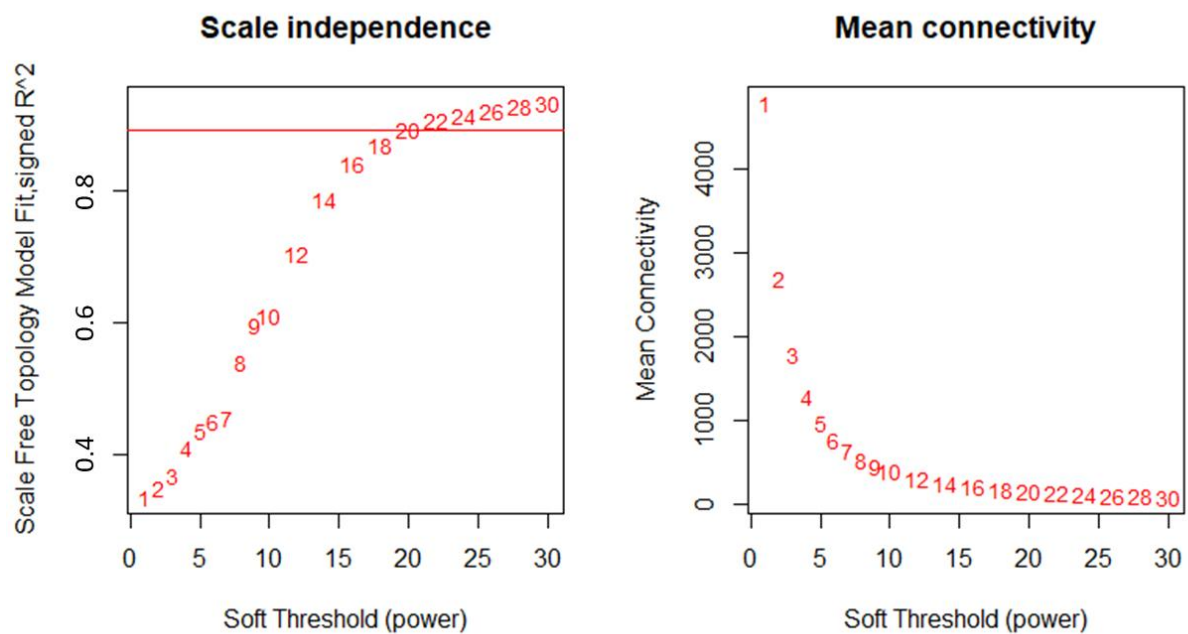

**Online Resource 4: Figure S1.** Determination of soft-thresholding power in the WGCNA.

Supplement: Supplementary file 1 [file DataSheet1.zip › Online resource 4.pdf]

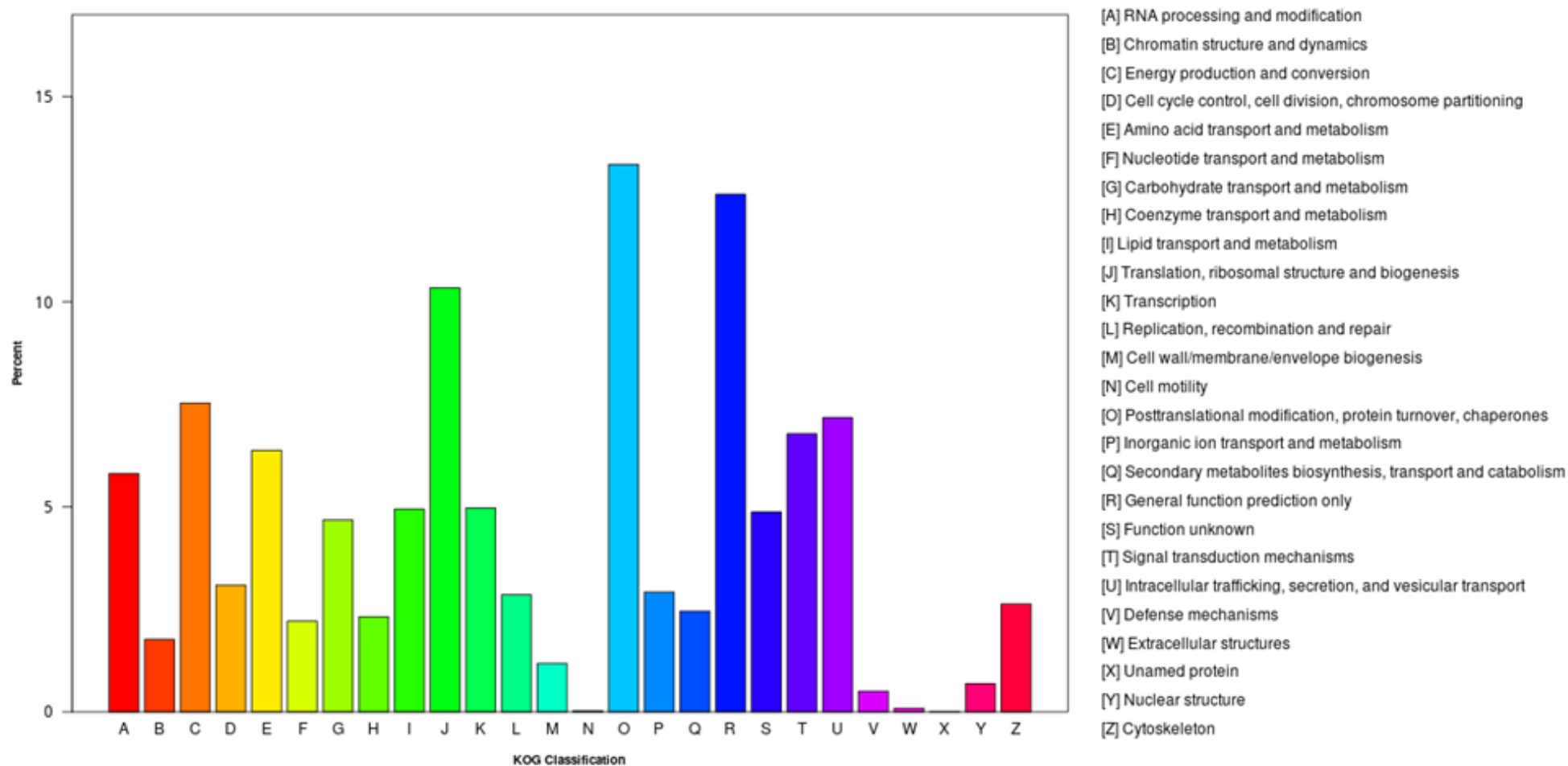

**Online Resource 5: Figure S2.** Unigenes KOG functional classification

Supplement: Supplementary file 1 [file DataSheet1.zip › Online resource 5.pdf]

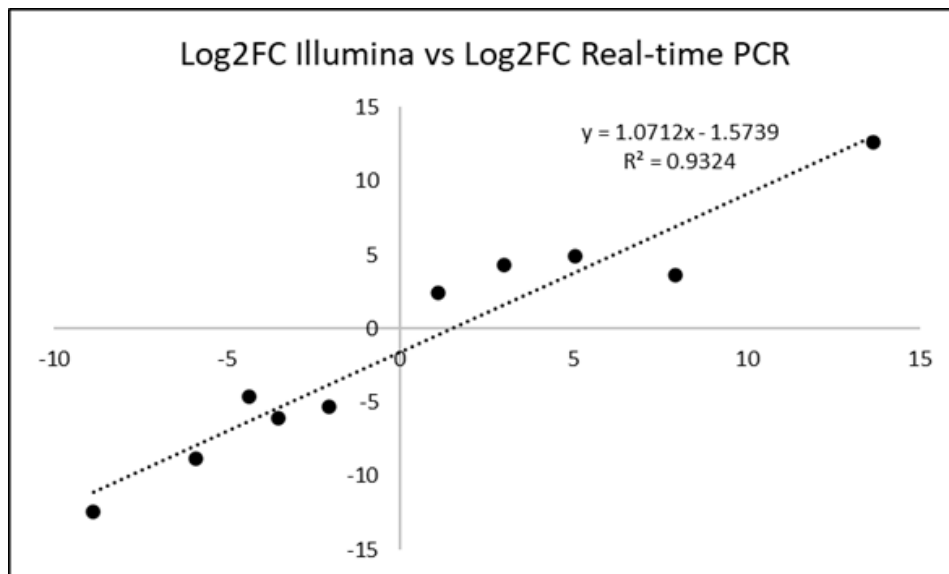

**Online Resource 6: Figure S3.** RNAseq validation

Supplement: Supplementary file 1 [file DataSheet1.zip › Online resource 6.pdf]
